# The role of toll-like receptors (TLRs) in pan-cancer

**DOI:** 10.1080/07853890.2022.2095664

**Published:** 2022-07-08

**Authors:** Runzhi Huang, Zehui Sun, Shuyuan Xian, Dianwen Song, Zhengyan Chang, Penghui Yan, Jie Zhang, Huabin Yin, Zixuan Zheng, Peng Hu, Zhenyu Li, Dan Huang, Yihan Liu, Chenyang Jiang, Man Li, Siqi Li, Tong Meng, Daoke Yang, Zongqiang Huang

**Affiliations:** a Department of Orthopedics, The First Affiliated Hospital of Zhengzhou University, Zhengzhou, China; b Division of Spine, Department of Orthopedics, Tongji Hospital affiliated to Tongji University School of Medicine, Shanghai, China; c Tongji University School of Medicine, Shanghai, China; d Department of Orthopedics, Shanghai General Hospital, School of Medicine, Shanghai Jiaotong University, Shanghai, China; e Department of Pathology, Shanghai Tenth People's Hospital, Tongji University School of Medicine, Shanghai, China; f Tongji University Cancer Center, Shanghai Tenth People's Hospital, Tongji University School of Medicine, Shanghai, China; g Department of Radiotherpy, The First Affiliated Hospital of Zhengzhou University, Zhengzhou, China

**Keywords:** Toll-like receptors (TLRs), multi-omics, pan-cancer, data-mining, tumour microenvironment (TME)

## Abstract

**Background:**

Toll-like receptors (TLRs) are important components of the innate and adaptive immune systems, and abnormal TLR expression has been linked to a variety of cancers. However, there was a lack of clarity on the association of TLR stimulation with the carcinogenesis of cancer. The study's goal was to analyse the clinical importance of TLRs expression at the mRNA level in pan-cancer datasets, as well as the link between TLR expression and carcinogenesis, progression, and clinical prognosis.

**Methods:**

The expression profile of TLRs derived from UCSC pan-cancer data was analysed in multiple dimensions, including clinical analysis, immunological subtype analysis, tumour microenvironment (TME) analysis, tumour stem cell correlation analysis, and drug sensitivity analysis. Additionally, we analyse protein-protein interactions, functional enrichment, and chromatin accessibility, as well as TLR expression in single-cell sequencing data.

**Results:**

Our multi-omics analysis results imply that TLRs may operate as a biological marker for carcinogenesis and progression, a potential target for anti-tumour therapy, and a prognostic biomarker, laying the theoretical groundwork for future translational medicine research.

**Conclusion:**

TLRs are involved in the formation of malignancies and can be explored in further detail as potential prognostic indicators.

**Key Messages**
- Toll-like receptors (TLRs) are key factors in the process of the innate and adaptive immune response, and their aberrant expression of TLRs have been widely reported in various cancer. However, the association between TLRs stimulation and tumorigenesis of cancer has not been well clarified.
- In this study, in the pan-cancer data, integrated TLR family gene expression analysis, clinical correlation analysis, immune subtype correlation analysis, tumour microenvironment correlation analysis, tumour stem cell correlation analysis, and drug sensitivity correlation analysis were performed.
- TLRs play an important role in the development of tumours and can be studied in depth as potential prognostic markers.

## 1. Introduction

TLRs are a type of pattern-recognition receptors (PRRs) expressed in immune cells. They are best known for their pathogen defense function, which involves the recognition of damage-associated molecular patterns (PAMPs) such as bacterial lipopolysaccharide and flagellin, as well as RNA produced during virus replication. TLRs, on the other hand, are involved in the identification of several endogenous ligands, including damage-associated molecular patterns (DAMPs) generated by dying or wounded cells [1]. The human body contains a total of 13 TLR genes, of which TLR1-10 are protein-coding genes and TLR11-13 are pseudogenes. Notably, unlike the majority of other TLR genes, TLR8-AS1 is an RNA gene that belongs to the lncRNA class. The TLR family is categorised into two subgroups based on their position: TLR1, −2, −4, −5, −6, and −10 on the cell surface, and TLR3, −7, −8, and −9 on the intracellular endosome [2,3]. TLR contains both external and intracellular domains as a type I transmembrane glycoprotein. The former contains the leucine repeat sequence and detects ligands selectively. The latter contains the Toll-interleukin 1 (IL-1) receptor domain (TIR) and activates downstream signalling pathways such as the NF-B, p38-MAPK, and JUN-kinase [4].

TLRs are broadly dispersed in numerous immune cells, including macrophages, dendritic cells (DC), neutrophils, B cells, epithelial and endothelial cells [5], as well as tumour-associated macrophages (TAMs) [6,7]. Apart from tumour-infiltrating immune cells, other tumour cells exhibit TLR activation [8,9]. TLRs can play a critical role in tumour initiation and progression by regulating the immune system, yet persistently activated TLRs can potentially produce a chronic inflammatory milieu and drive carcinogenesis [10].

Given that genes in the TLR family have distinct ligands and downstream mechanisms of action in various malignancies, the precise activities of several of these genes remain unknown [11]. Considering TLR's unique double-edged sword effect of being both pro- and anti-tumour, it is worthwhile to do a comprehensive investigation of TLR expression in pan-cancer. The expression of TLR in pan-cancer was discovered in this investigation. Additionally, we explored the correlation between TLR genes and clinical overall survival (OS), tumour immune subtype, interstitial microenvironment, tumour stem cells, and drug sensitivity using a multi-omics analysis at the genomics, transcriptome, and proteome levels, as well as preliminary experimental validation. The findings demonstrated a strong correlation between TLRs and tumour growth and clinical prognosis, emphasising the importance of additional translational medicine research.

## 2. Materials and methods

### 2.1. Data downloading and interpretation

Our analysis is based entirely on data from existing databases. The mRNA sequencing data, clinical data (including phenotype and overall survival), immunological subtype, and stemness score information for 33 types of tumours [12] and paired adjacent tissues (a total of 11,057 samples) were retrieved from the UNSC Xena website (http://xena.ucsc.edu/). Among the above, the mRNA level expression was quantified using Fragments per Kilobase of transcript per Million mapped reads (FPKM), and survival data were downloaded from TCGA cancer datasets collected by Genomic Data Commons (GDC) (version, 07-19-2019), whereas the phenotype information was obtained from GDC-collected TCGA cancer datasets (version, 08-07-2019). Additionally, we downloaded immunological subtypes and stemness scores assessed by DNA methylation and RNA expression from the TCGA Pan-Cancer (PANCAN) datasets (version, 2018-04-03). After translating the ensemble ID to the gene symbol (official name) in R, the expression profiles of TLR1, TLR2, TLR3, TLR4, TLR5, TLR6, TLR7, TLR8, TLR9, TLR10, TRL12P, and TLR8-AS1 were retrieved for further study.

Additionally, we downloaded all ATAC-seq count matrices for cancer types from the GDC data portal (https://gdc.cancer.gov/about-data/publications/ATACseq-AWG), which included 23 TCGA cancer types (Table S2), 410 tumour samples, 796 chromatin accessibility atlases, and 562,709 transposase-accessible DNA elements [13].

### 2.2. Differentially expressed TLR genes analysis and correlation analysis

We used differential expression studies with the Wilcox test to elucidate the general rule of transcriptome expression in pan-cancer. To assure the study's significance, the analysis process included only cancer species with at least two adjacent specimens (23 of the 33 TCGA cancers). Besides, FC (fold change) values of TLR expression level between paired samples were obtained for further detection. Then, we utilised boxplots and a heatmap to illustrate the differences in TLR expression between cancer and surrounding tissues: The colour in the heatmap denotes log2FC value. Colour red means that the gene is up-regulated in the tumour, Colour blue shows that the gene is down-regulated, and colour white indicates that there is no variation in expression levels. In general, a TLR expression level with a log2FC value > 1 or < −1 was considered differentially expressed. In view of the putative regulatory relationship between genes of the same family, we ran liner correlation analysis with *t* test on the expression level of TLR genes in pan-cancer data. The significance level was established at two pairs *p* < .05. Correlation and significance values were displayed in the heatmap as correlation coefficients, colours, and sizes of dots.

### 2.3. Clinical correlation analysis of TLR genes

The Kaplan-Meier method was utilised to generate survival curves of patients with above and below the median TLR expression, respectively, using follow-up time as the horizontal axis and survival rate as the vertical axis in a study of 33 cancer patients. We also used the log-rank test to look for any significant changes in the factor of survival time (two pairs *p* < .05). The COX proportional hazard model with *χ*2 test was carried out to perform multi-factor analysis and to evaluate effected TLR genes on the prognosis using hazard ratio. Furthermore, liner correlation analyses of TLR expression with clinical outcomes such as tumour stage, grade, and recurrence were conducted. When the *P* value on both sides was less than .05, the difference was declared statistically significant.

### 2.4. Immune type correlation analysis of TLR genes

In pan-cancer research, the immune microenvironment characteristics were used to classify tumour tissues into immune subtypes, which are currently classified as Wound Healing (Immune C1), IFN-gamma Dominant (Immune C2), Inflammatory (Immune C3), Lymphocyte Depleted (Immune C4), Immunologically Quiet (Immune C5), and TGF-beta Dominant (Immune C6) [14]. We used the Kruskal test to identify differentially expressed TLR genes in six distinct immunological subtypes in order to determine if TLR genes have an effect on immune infiltration patterns. *p* < .05 was considered significant for two pairs.

### 2.5. Tumour microenvironment correlation analysis of TLR genes

The microenvironment scores were calculated using the ESTIMATE (Estimation of Stromal and Immune cells in Malignant Tumour tissues Using Expression Data) algorithm [15]. Three scores were applied to measure microenvironment data: stromal score, immune score, and estimation score. The stromal score indicates the number of stromal cells (fibroblasts and vascular endothelial cells) in tumour tissues; the immune score indicates the number of immune cells (T cells and B cells); and the estimated score indicates the total of the stromal and immune scores. A higher estimate score suggests that the tumour is less pure. Spearman correlation analysis was used to determine the relationship between the expression levels of TLR genes in 33 tumour tissues and the three scores. And the results were shown using heatmaps.

### 2.6. Tumour stemness correlation analysis of TLR genes

Cancer stem cells (CSCs) are tumour cells that have the ability to self-renew and differentiate into different types of tumour cells. Typically, these cells are identified as having the potential to promote tumour formation, progression, recurrence, and resistance to treatment. Stemness scores for tumour samples are generated using DNA methylation (DNAss) and mRNA expression (RNAss) data. They range from 0 to 1 and are calculated using the one-class logistic regression (OCLR) technique [16]. In general, a higher score suggests more stem cell characteristics. Similarly, Spearman correlation analyses were used to determine the relationship between the expression of TLR genes and the stemness scores of each type of cancer. Heatmaps were used to display the results.

### 2.7. Drug sensitivity analysis of TLR genes

Cell Miner database (version, 2.2, https://discover.nci.nih.gov/cellminer/) offered processed datasets of matched mRNA sequencing and compound activity data (NCI-60 cell line sets maintained by the National Cancer Institute of the United States of America) for drug sensitivity study [17,18]. The z score in a compound's activity profile indicates the cell's sensitivity to the medication. The greater the value, the more potent the drug's anticancer activity. Pearson correlation analysis was used to determine the relationship between the level of TLR gene expression and the z score of each chemical. It should be highlighted that the correlation analysis included only FDA-approved medications and clinically validated substances.

### 2.8. Tmb correlation analysis and genomic alteration of TLR family in pan-cancer

Tumour mutation burden (TMB) is defined as the total number of non-synonymous mutations inside the exon coding region per megabase of the genome studied. Currently, it is considered that the greater the TMB value, the more types and numbers of neoantigens created by the tumour, and thus the greater the probability of being recognised by the immune system. As a result, tumour patients with a high TMB (TMB: >20 mutations/mb) are more susceptible to immune therapy, such as PD-1/PD-L1 inhibition [19]. A thorough analysis of TMB in 33 different cancer types was conducted in order to characterise the mutation in the form of log10 (TMB + 1), using an algorithm described by Lawrence et al. [20]. Following that, Pearson correlation analyses were performed between TMB and TLR family mRNA expression levels.

Moreover, we used the cBioportal browser (version, 3.4.16, http://www.cbioportal.org) [21] to identify TLR family genomic variants in a pan-cancer analysis, with an onco-print illustrating the mutation spectrum across several malignancies.

### 2.9. Interaction of TLR family at the protein level

The first step was to determine the overall protein level expression of TLR family members in cancer and neighbouring normal tissues using immunohistochemistry (IHC) results from the Human Protein Atlas database (version, 19.3, https://www.proteinatlas.org/) [22]. To determine the interaction of TLRs at the protein level, an online bioinformatic analysis of the proteome and RPPA data from diverse tumour types was performed using the LinkedOmics browser (www.linkedomics.org/) [23].

### 2.10. Functional enrichment analysis of TLR family and pathway-level analysis in pan-cancer

We began by annotating TLR genes using GO (Gene Ontology, version, 10.5281/zenodo.3954044, available at http://geneontology.org/) [24] and KEGG (Kyoto Encyclopaedia of Genes and Genomes, Version, 95.0, https://www.genome.jp/kegg/) function enrichment analysis [25] to determine the location, molecular function, and biological process of gene products, as well as to examine the signalling pathways linked with TLRs.

Then, using the methodology described by Sanchez-Vega et al., we analysed the prevalence of somatic mutations in canonical pathways to validate the pathway modification linked with the TLR family across different tumour types [12]. The 33 TCGA cancer types were subdivided into 64 genomically distinct tumour subtypes, and both pathway members (oncogene activating events and tumour repressing gene inactivating events) and individual alterations (statistical recurrence and presumed function impact) of key genes defied as functional importance were included in the analysis. To further validate the association between the TLR family and signalling, we created a protein-protein interaction (PPI) network using the String database (Version, 11.0 b, https://string-db.org/) [26].

### 2.11. Expression of TLR family in single-cell genomics

Because high-throughput mRNA sequencing employs tissue samples made up of a mixture of millions of cells, the results represent an average of gene expression, reflecting only the expression pattern in the numerically dominant cell population. In comparison to conventional mRNA sequencing, single-cell RNA sequencing (scRNA-Seq) generates an individual genetic profile for each cell and identifies uncommon cells from diverse tumour samples, allowing for the exploration of features specific to a single cell. During this process, individual cells are isolated, their transcripts are captured, and sequence libraries are generated, as well as individual cells are mapped to the transcripts.

In our study, we used scRNA-Seq data from various common tumour tissues, including colorectal cancer (Accession no. E-MTAB-8410) [27] (Accession no. E-MTAB-8410), lung cancer (Accession no. E-MTAB-6308) [28], and head and neck squamous cell carcinoma (Accession no. SRP226817) [29], which were collected by the Single Cell Expression Atlas database (version, 12-October-2020, https://www.ebi.ac.uk/gxa/sc/release-notes.html) [30]. Results were visualised as t-SNE figures.

### 2.12. Chromatin accessibility analysis of TLR family

The Assay for Targeting Accessible-Chromatin with High-Throughput Sequencing (ATAC-Seq) technique [31] is a relatively new method for detecting accessible chromatin at the genome-wide level. In this section, we downloaded and arranged ATAC-Seq peak counts matrices for various cell lines. These matrices were then combined with the TLR mRNA expression matrix in R. We annotated the data with the names of the sequenced chromosomes, starting and finishing sites, genomic characteristics, ensemble ID, and gene name after Pearson correlation analysis between peak counts of a certain chromosomal region and mRNA expression level. The graphic images depicted the link between TLR gene sites and peak counts within each cell line.

### 2.13. Statistical analysis

We used Perl (Practical Extraction and Report Language, https://www.perl.org), R software (version 3.6.1, http://www.r-project.org; Institute for Statistics and Mathematics, Vienna, Austria), and Bioconductor packages (http://www.bioconductor.org/packages/release/bioc/html/impute.html) to further process and analyse raw data. And a *P* value less than .05 for the two tails was considered significant.

## 3. Result

### 3.1. The differentially expressed TLR genes and their co-expression in pan-cancer

**Figure 1.**
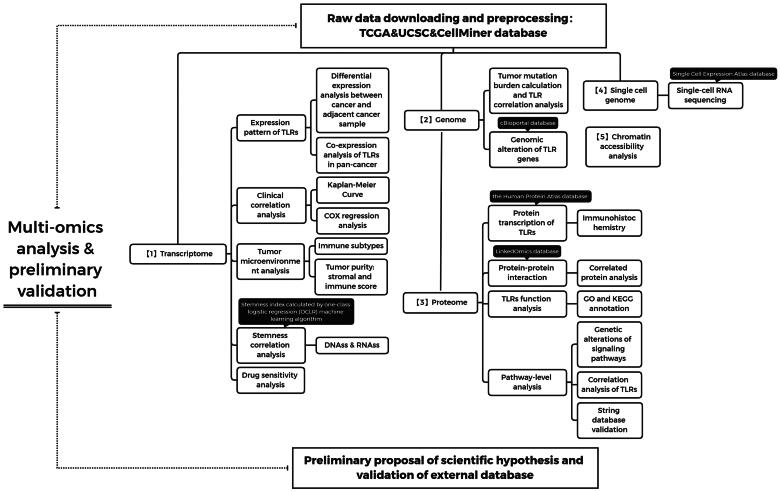
Flow chart.

**Figure 2.**
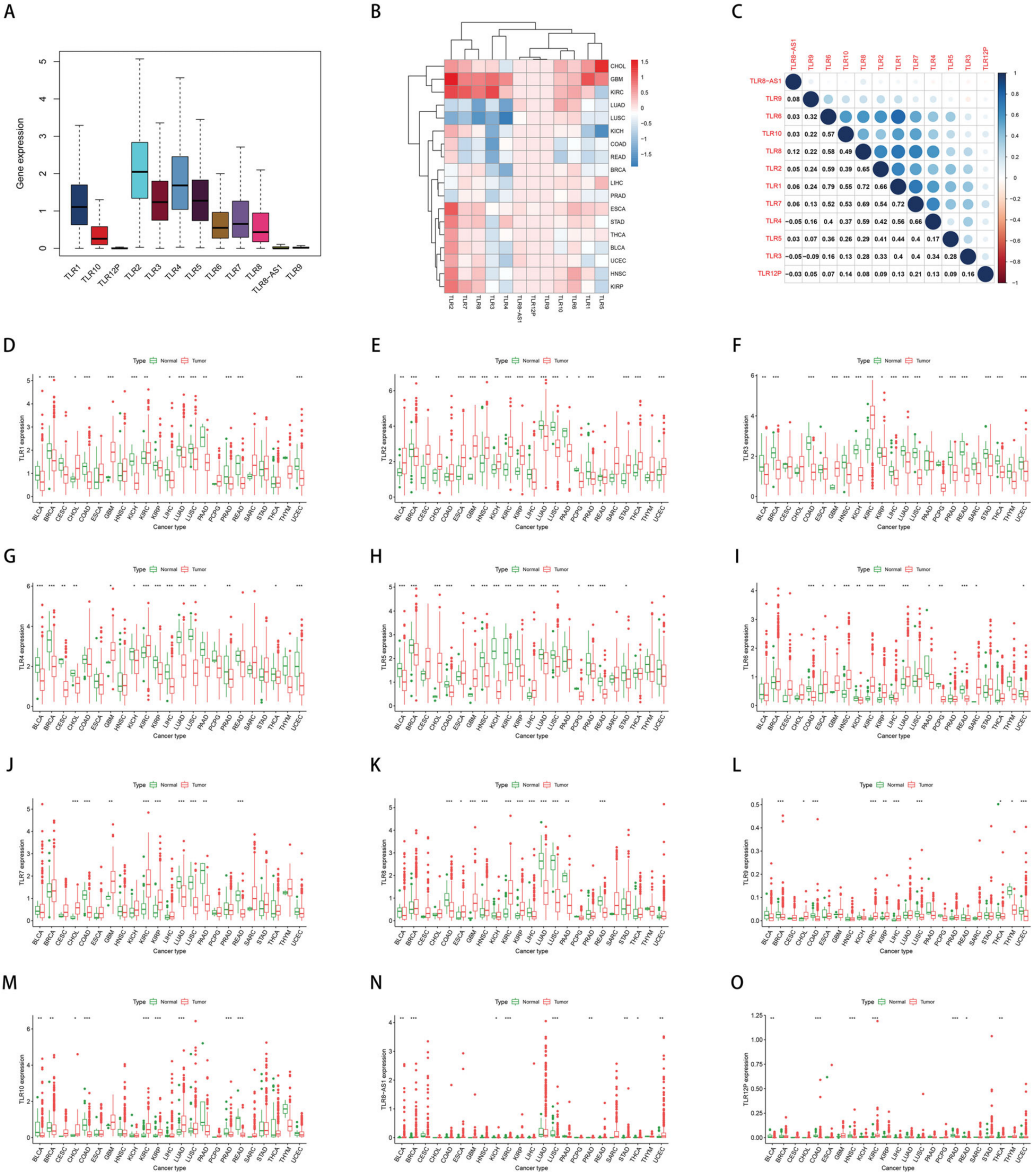
Differentially expressed TLR genes analysis and correlation analysis in various cancers. (A) Expression profile of TLR genes in pan-cancer. (B) Differential expression analysis: Red indicates that the gene is up-regulated in the tumour; blue indicates down-regulated; and white indicates no difference in expression levels. The density represents the log2 (fold change) value. (C) Co-expression analysis between TLR family. (D) The expression level of each TLR gene in different cancer.

The entire process of this study is depicted in Figure 1, and TLR gene expression profiles are included in Figure 2. As reported in Figure 2A, TLR2 and TLR4 were found to be overexpressed; TLR3 and TLR5 were at medium levels; TLR6, TLR7, TLR8 and TLR10 were at low levels. Additionally, TLR9, TLR8-AS1, and TLR12P were infrequently detected in these cancer tissues.

We discovered that the majority of TLR genes were usually up- or down-regulated in different cancer tissues to varying degrees based on differential expression analyses in cancer and surrounding tissues in 23 cancer types. TLR2 and TLR8-AS1 expression was increased in the majority of tumours, although TLR2 expression was decreased in LUSC (P0.001), LUAD (P0.001), PRAD (P0.001), BRCA (P0.001), and LIHC (P0.001), and TLR8-AS1 expression was specifically decreased in LUSC (P0.001) and PRAD (P0.001). TLR1, TLR3, TLR4, TLR5, and TLR12P were all down-regulated in the majority of malignancies. TLR3 was not up-regulated in any of the cancer tissues tested, whereas TLR1 was inversely up-regulated only in KIRC (P0.01), CHOL (P0.05), and GBM (P0.001). Only KIRC (P0.001) and GBM (P0.05) demonstrated increased TLR4 expression. TLR5 expression was significantly increased in LIHC (P0.001), GBM (P0.01), and CHOL (P0.001). TLR12P transcriptional expression was significantly increased in THCA (P0.01) and KIRC (P0.001). Additionally, TLR6, TLR7, TLR8, TLR9, and TLR10 expression was up- or down-regulated in different cancer species (Figure 2B, D).

Additionally, there is a generally positive connection between the co-expression of TLR genes, demonstrating that TLR genes have a universal co-expression relationship (Figure 2C). Notably, there are some strong correlations between TLR1 and TLR6 (*R* = 0.79), TLR1 and TLR7 (*R* = 0.72), TLR1 and TLR8 (*R* = 0.72), TLR7 and TLR8 (*R* = 0.69), and TLR1 and TLR2 (*R* = 0.66), showing that those genes share some type of expression or functional relationship. Given that TLR4 and TLR6 have been shown to form heterodimers, our co-expression data corroborated the relationship [26].

### 3.2. Clinical correlation analysis of TLR genes

**Figure 3.**
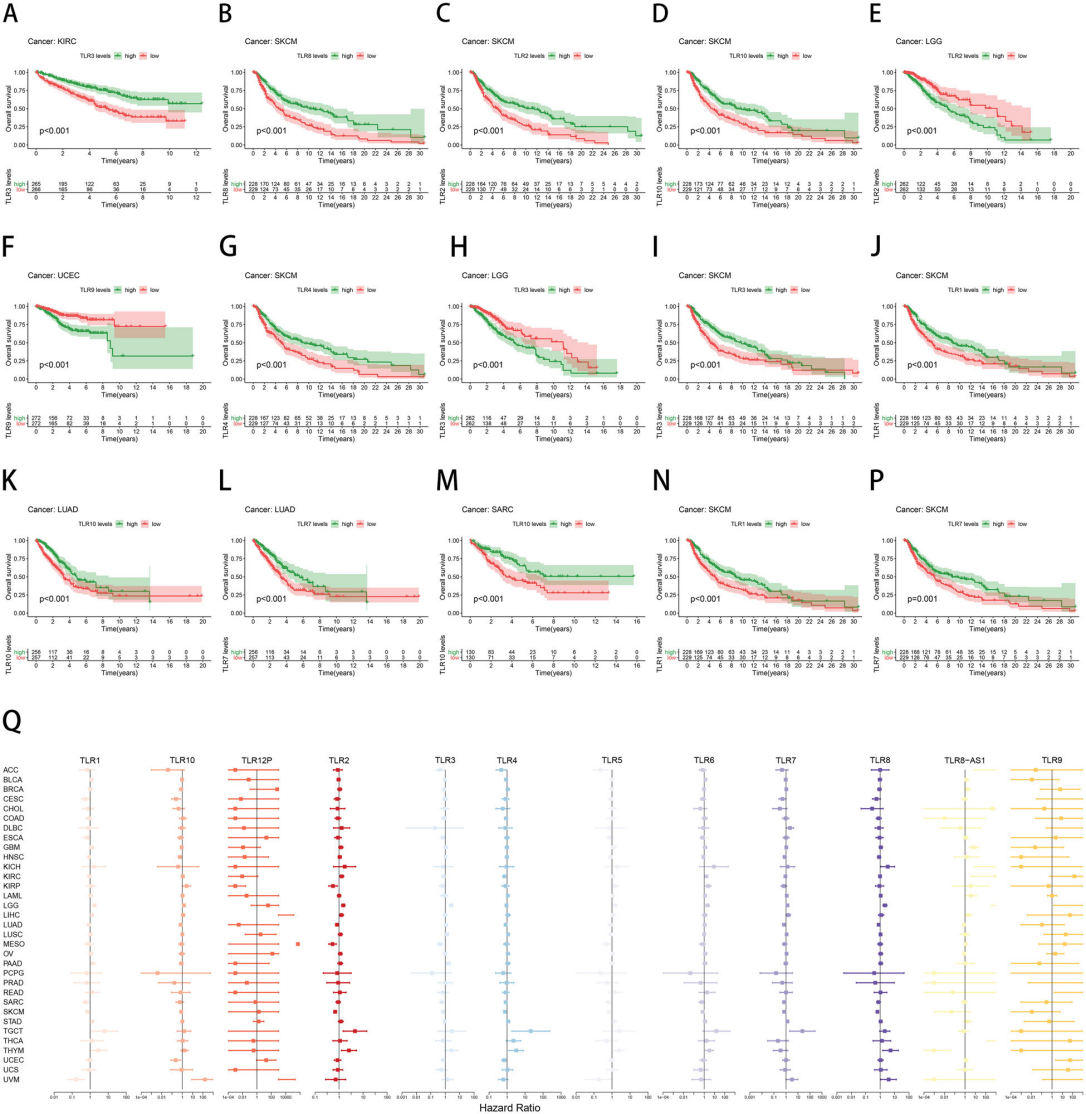
Clinical correlation analysis of TLR genes in pan-cancer. (A–P) Survival analysis of TLR genes shown as K-M curves. (Q) COX regression analysis of TLR genes.

Kaplan-Meier and COX regression analysis demonstrated that all TLR genes, at the mRNA level, were capable of classifying patients into groups with a good or bad prognosis in at least three forms of cancer. The 15 survival analysis results with the lowest P values are depicted in Figure 3A, alphabetically by gene name. Indeed, determining the precise effect of TLR expression on the clinical fate of cancer patients has been difficult, as high expression of a particular TLR in different cancer types might be associated with a favourable prognosis or can also result in a poor outcome.

For example, high TLR2 expression in SKCM (*p* < .001) indicated a longer survival duration for patients, whereas low TLR2 expression in LGG (*p* < .001) suggested a shorter survival period for patients. Additionally, increased TLR3 expression was related to a favourable prognosis in KIRC (*p* < .001) and SKCM (*p* < .001) samples, but not in LGG (*p* < .001).

On the one hand, TLR3, TLR4, and TLR10 were the most strongly associated with survival within the TLR family. TLR3 and TLR10 were found to be significant in nine malignancies, while TLR4 was shown to be significant in eight cancers. TLR5, TLR7, and TLR8-AS1 were found to be significant in seven cancers each. On the other hand, TLR gene expression had the greatest effect on the prognosis of SKCM, LGG, and KIRC. Additionally, nine TLR genes were shown to be substantially associated with the prognosis of SKCM patients, eight with the prognosis of LGG patients, and seven with the prognosis of KIRC patients. In a nutshell, the results above revealed that TLRs, particularly TLR3, TLR4, and TLR10, had the potential to serve as clinical prognostic indicators.

Cox proportional hazards regression was used to further investigate the relationship between TLR and clinical outcomes (Figure 3B). Among these, TLR8-AS1 expression was associated with a favourable prognosis in up to seven malignancies, including LAML (HR = 3.86, *p* < .05), GBM (HR = 7.07, *p* < .001), KIRC (HR = 1094.82, *p* < .05), ACC (HR = 3928.22, *p* < .01), THCA (HR = 996958.39, *p* < .01), LGG (HR = 27894655.28, *p* < .01), and KICH (HR = 92551036.36, *p* < .05). This occurrence may be related to the lncRNA's regulatory functions. TLR7 expression was found to be a significant risk factor for six different forms of cancer: BLCA (HR = 1.33, *p* < .05), KIRC (HR = 1.66, *p* < .01), LGG (HR = 2.17, *p* < .001), KIRP (HR = 2.52, *p* < .01), and THYM (HR = 3.23, *p* < .05). Additionally, TLR2 and TLR6 expression were found to be poor predictive markers for five distinct forms of cancer, respectively.

Following that, we conducted investigations of the connection between TLR expression and clinical events in order to further establish the clinical relevance of TLR family expression levels. In terms of clinical stages, we discovered that the expression levels of multiple TLR family members, including TLR3, TLR4, TLR8, TLR10, and TLR8-AS1, were significantly different between stage I and IV of BRCA, COAD, HNSC, and STAD (Figure S1), implying the involvement of the TLR family in tumour progression mechanisms, which may have implications for diagnosis and clinical risk prediction.

### 3.3. Immune subtype analysis of TLR genes

To a certain extent, the immunological subtype of cancer is strongly associated with its incidence, progression, and clinical prognosis. We discovered that TLR gene expression levels varied considerably between the six immunological subtypes of pan-cancer (*p* < .001). TLR1, TLR2, TLR3, TLR4, and TLR5 expression levels were high across the six immune subtypes in the pan-cancer data; TLR6, TLR7, TLR8, and TLR10 expression levels were moderate; and TLR9, TLR12P, and TLR8-AS1 expression levels were extremely low. In general, the levels of expression of various genes were relatively high in C6. Figure S2A).

TLR gene expression varied significantly among the six immunological subtypes found in various cancer types. For instance, whereas the expression trend in LUAD was generally identical to that in pan-cancer, TLR9 was not differentially expressed amongst any of the six immunological subtypes. Additionally, TLR2 gene expression was significantly higher in C3 and C4 (*p* < .001), and the overall expression level of TLR8-AS1 also increased (*p* < .05). TLR gene integral expression levels were lower in LIHC, SARK, and SKCM, indicating that TLR genes were substantially expressed in C2 or C6 but were absent in C1 (Figure S2C–5E). Given that the TLR family is associated with immune-related genes, we hypothesised that detecting TLR expression fingerprints across different cancer types or samples could aid in developing more accurate and effective immunotherapies.

### 3.4. Tumour microenvironment analysis of TLR genes

In addition to tumour cells, there are immune and stromal cells in tumour tissue, which form the micro-environment, and constant communications between tumour and non-tumour cells jointly regulate the growth of neoplasm. The association between TLR gene expression levels and the stromal score, immunological score, and estimation score was examined in 33 TCGA tumour samples. It may allow us to better understand their involvement in the tumour microenvironment and purity, which were found to be substantially connected with clinical, genetic, and biological aspects of individuals with malignancies. Almost all malignancies had a substantial positive correlation between TLR gene expression and the number of interstitial cells, most notably in the study results for TLR1, TLR2, TLR4, TLR6, TLR7, TLR8, and TLR10 genes (Figure S3), consistent with the PRRs function of the TLR family.

### 3.5. Stem cell characteristics analysis of TLR genes

In this study, we employed stemness indices such as RNAss (derived from mRNA expression) and DNAss (derived from DNA methylation signature) to evaluate stemness traits that indicate self-renewal and dedifferentiation potential. Due to the fact that RNAss and DNAss use distinct input files, their findings may be inconsistent. Correlation examination of TLR expression and stemness index, on the other hand, demonstrated that both RNAss and DNAss had a high degree of connection with TLRs for the majority of malignancies.

TLR gene expression was significantly negatively associated with RNAss (Figure S4A) in BLCA, BRAC, CESC, COAD, DLBC, ESCA, KIRP, LGG, LUAD, LUSC, READ, and STAD tumours, implying that tumours with high TLR expression exhibited a higher degree of differentiation and lacked cancer stem cell characteristics. It's worth noting that TLR1, TLR2, TLR3, TLR7, TLR8, and TLR9 were adversely connected with RNAss in the majority of cancers, but favourably correlated with RNAss in a very small number of tumours (KIRC, MESO, LAML, CHOL, KICH and THYM). Additionally, TLR8-AS1, a lncRNA, demonstrated a positive connection with RNAss in ACC, CHOL, SARC, TGCT, and THYM, which was inconsistent with the expression trends of other TLR genes. Given this, we hypothesised that TLR1, TLR2, TLR3, TLR7, TLR8, and TLR9 would be involved in a negative regulatory mechanism mediated by TLR8-AS1 that contributes to the maintenance of cancer stem cell features.

**Figure 4.**
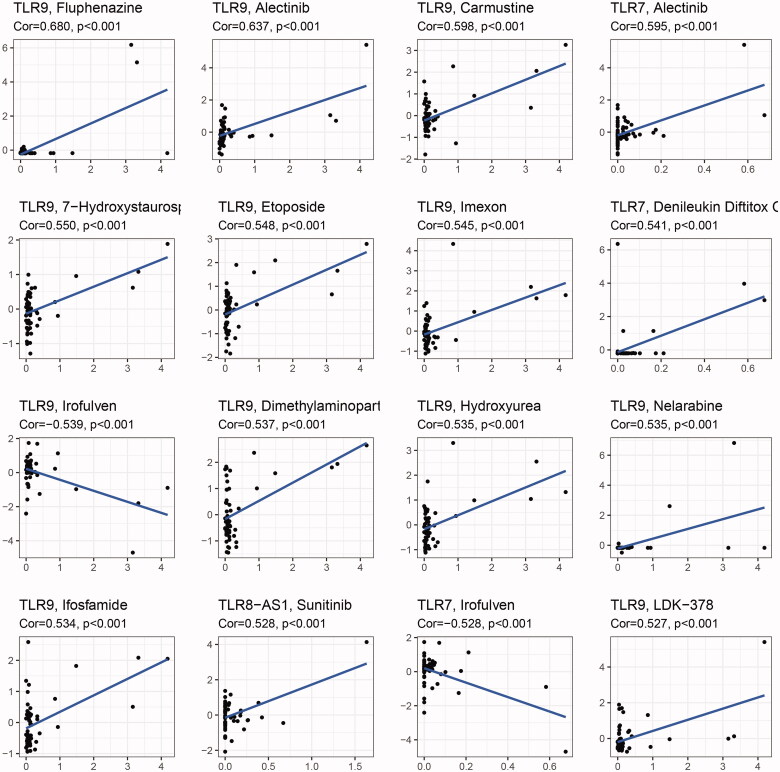
Drug sensitivity analysis of TLR genes.

Although there was a considerable association between TLR family expression and DNAss in cancers, the positive or negative correlations between 33 tumours were quite diverse (Figure S4B). DNAss analysis revealed that while high TLR gene expression was adversely connected with stem cell features in the majority of malignancies, it was positively correlated in a few tumours (ACC, CHOL, KIRP, LAML, LGG, PCPG, PRAD, THCA, THYM, and UVM). As a result, the association between TLRs and DNAss cannot be established definitively.

Additionally, we examined the link between TLR family genes and the intratumor microenvironment and stemness index in single tumour data from BRCA, COAD, HNSC, and LIHC (Figure S5), and the correlation pattern remained consistent with the pan-cancer trend. TLR4 and TLR7 were the most strongly correlated with tumour stemness features, but TLR9 had an undefined connection with the stemness index in various malignancies, and TLR4, TLR7, and TLR8 were the most associated with the tumour microenvironment. The verification results above were consistent with the findings of the study on pan-cancer.

**Figure 5.**
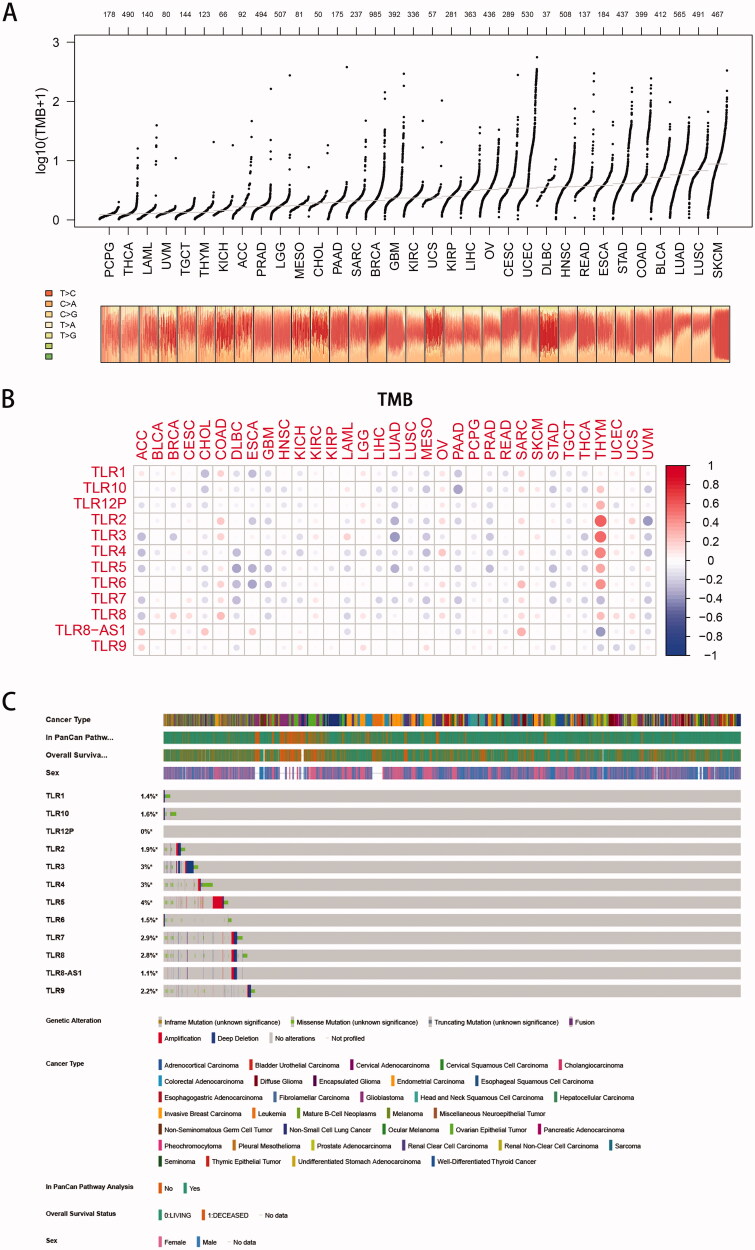
Tumour mutation burden analysis. (A) Overall view of tumour mutation frequency of each cancer. (B) Correlation analysis between TLR genes and tumour mutation burden (TMB): the colour and density of the dots represent the positive or negative correlation and the value of the correlation coefficient. (C) An onco-print plot shows the genomic alterations of TLR genes in pan-cancer, containing the genetic alteration, cancer type, and overall survival status information.

### 3.6. Drug sensitivity analysis of TLR genes

For correlation analysis, the data on drug sensitivity (z-score) of the various lines of cancer cells derived from the Cell Miner database and the TLR family of gene expression were employed. And Figure 4 displays the 16 results with the highest correlation coefficients, sorted by Cor value. The investigation revealed that the degree of expression of all ten protein-coding genes in the TLR family can alter tumour cells' sensitivity to certain medications. Among them, TLR9 was the most highly linked gene with drug sensitivity, and it also influenced the activity of the most drug types (up to 81). TLR9 expression was positively linked with sensitivity to the majority of inhibitors, implying that patients with high TLR9 expression may be more susceptible to anti-tumour treatment. However, it is worth mentioning that TLR7 and TLR9 expression levels were negatively linked with cancer cell sensitivity to Irofulven, an alkylating agent, indicating that as TLR7 and TLR9 expression levels grew, the risk of Irofulven resistance increased. We hypothesised that this occurrence could be explained by the positive association between TLR7, TLR9, and DNAss, although the specific mechanism required additional investigation.

### 3.7. Correlation of TLR family and TMB in pan-cancer

The TMB (in the form of log10 (TMB + 1)) of 33 TCGA tumours was plotted in Figure 5A, from smallest to greatest in terms of the median frequency of somatic mutations. As illustrated in the image, SKCM, LUSC, LUAD, and BLCA were all malignancies with a significant mutation rate. Among them, ultraviolet radiation is a clear mutagenic factor in SKCM, smoking is clearly associated with the development of lung cancer (squamous cell carcinoma and adenocarcinoma, respectively), and unhealthy eating habits such as drinking are strongly associated with the appearance of gastrointestinal tumours such as COAD, STAD, and ESCA. However, malignant disorders of the neurological and endocrine systems, such as PCPG and THCA, had lower TMB levels, implying that environmental features had some influence on the occurrence of oncogenic mutations.

Co-expression analysis (Figure 5B) revealed that, whereas TLR was typically negatively connected with mutation levels in 33 cancers, a few malignancies (such as COAD, OV, THYM, and SARC, etc.) were predominantly positively correlated. Additionally, the association between the TLR family genes and TMB remains unknown, but the majority of them showed a negative correlation with TMB, except for TLR9.

Additionally, as illustrated in Figure 5C, genes in the TLR family have low-level but rather prevalent genomic alterations in a variety of malignancies. TLR5 had a mutation frequency of up to 4%, primarily amplification, whereas TLR3 had a mutation frequency of 3%, with deep deletion being more prevalent, and TLR4 had a mutation frequency of 3%, with the majority of missense variants. Additionally, the content above indicated that mutant TLRs potentially act as tumour driver genes.

### 3.8. Interaction of TLR family at the protein level

**Figure 6.**
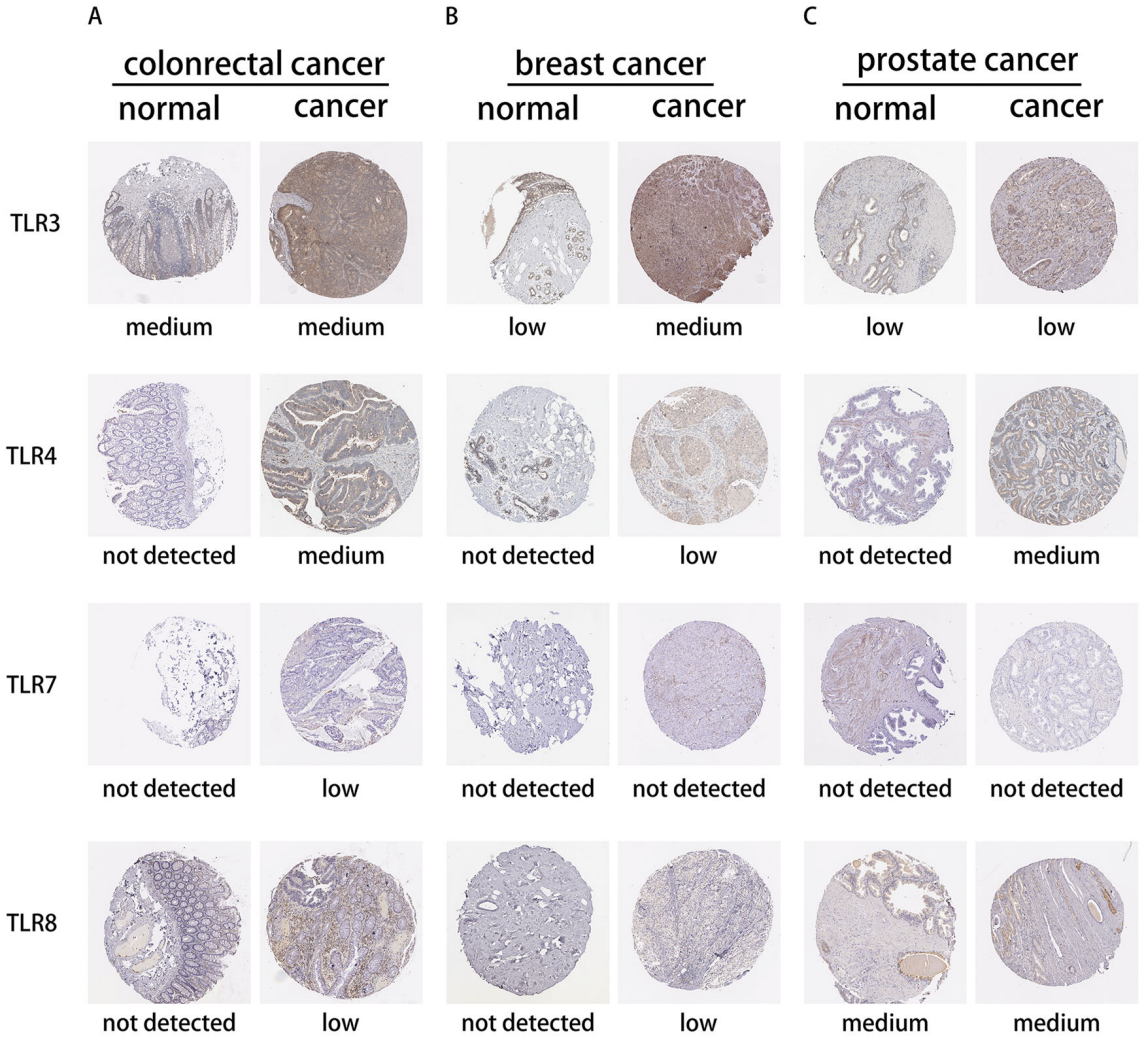
The expression level of TLR3, TLR4, TLR7, and TLR8 in the proteomics level.

To begin, we determined the TLR family's protein expression using the Human Protein Atlas database. Due to a lack of TLR family immunohistochemical samples, only TLR3, TLR4, TLR7, and TLR8 staining results in colorectal cancer, breast cancer, prostate cancer, and normal tissue were exhibited (Figure 6), which were largely consistent with the previously reported mRNA expression. Following that, proteomic research of several cancers indicated that TLRs interacted with a variety of proteins implicated in tumour-linked pathways such as RTK/RAS, TP53, NF-B, WNT, MYC, and cell cycle pathways, etc. (See Figure S6).

### 3.9. Functional enrichment analysis of TLR family and pathway-level analysis

**Figure 7.**
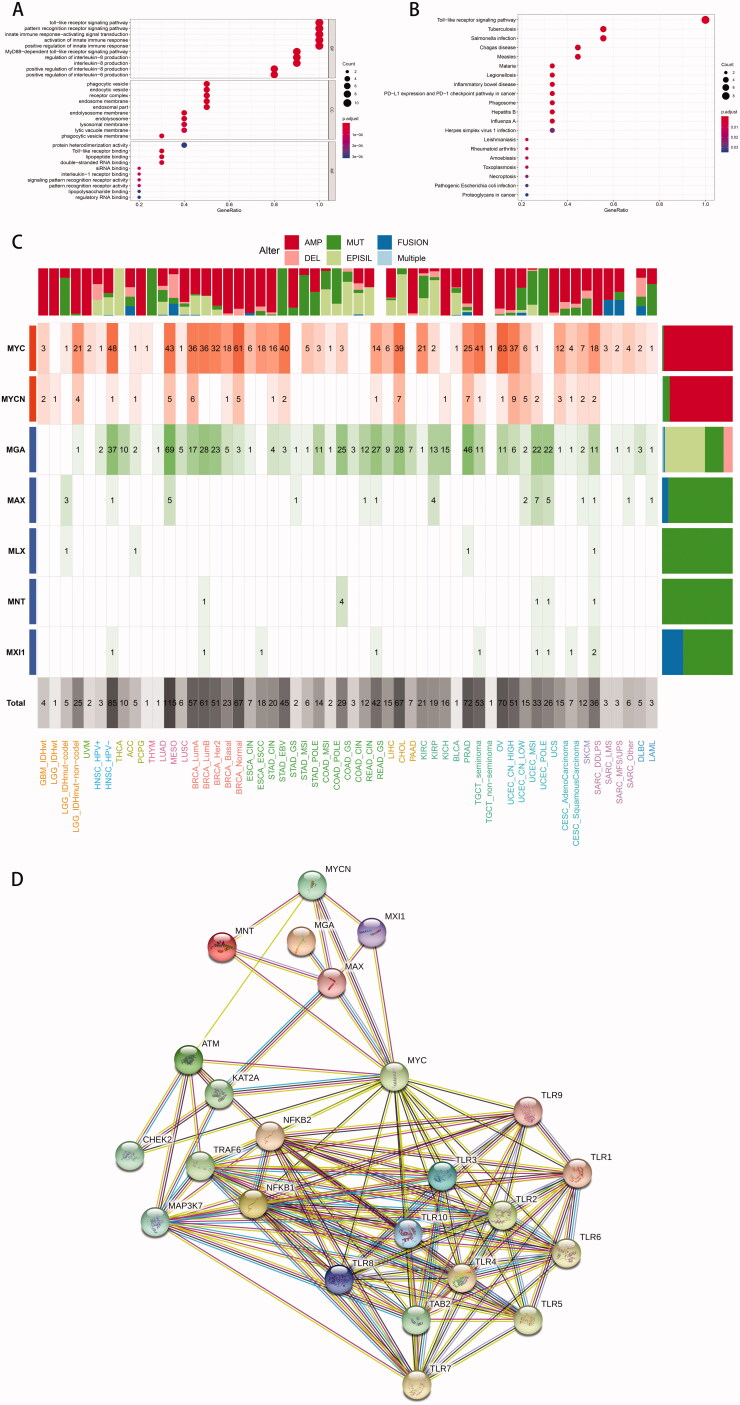
Functional enrichment analysis of TLR family. (A) GO-term function enrichment analysis. (B) KEGG pathway enrichment analysis. (C) C-MYC pathway alterations: a shade of dark red refers to alterations of amplification, and pink represents deletion. The dark and light green implies the mutation and methylation respectively. And the fusion and multiple genes are shown as a dark and light blue bar. (D) Protein and protein interaction network between TLR family and molecules associated: each line represents a connection between two proteins.

The functional enrichment analysis (Figure 7A, B) was performed to confirm the TLR family's universal function in various cancers, noting that TLRs were closely related to a variety of other cancer-related biological processes, such as PD-L1 expression and the PD-1 checkpoint pathway, except for immune and inflammation response.

As a large number of studies reported that TLR blockade altered the activity of c-MYC [32–39], a protein is known to be involved in the regulation of cell cycle progression, differentiation, apoptosis and metabolism, we hypothesised that the c-MYC pathway was involved in the TLR family's tumour-promoting mechanism. Taking into account the possibility of carcinogenic events associated with frequent genetic modifications in signalling pathways, we determined the amount, mechanism and co-occurrence of mutations in the c-MYC pathway across various tumour types and subtypes, as depicted in the heatmap (Figure 7C).

MYC, MYCN, MGA, MAX, MLX, MNT, and MXI1 were the genes examined in the c-MYC pathway, where mutations in MYC and MYCN were defined as oncogenes, and mutations in other genes were characterised as tumour suppressor genes. Clearly, MYC was the most frequently changed gene across many tumour types, followed by MGA. MYC and MYCN had the largest frequency of amplification, while MGA had the highest frequency of epigenetic silencing, whereas other genes were primarily mutated. MYC mutations were frequent in gynaecologic cancers (high CN UCEC, 63%), breast cancers (normal BRCA, 61%), and HPV-negative head and neck cancers (NHSC HPV-, 48%). Furthermore, MGA mutations were most frequently seen in lung cancer (LUAD, 69%), prostate cancer (TGCT seminoma, 46%), and HPV-negative head and neck cancer (TGCT seminoma, 46%). (NHSC HPV-, 37%). On the other hand, lung adenocarcinoma (LUAD, 115%) exhibited the greatest cumulative c-MYC pathway change frequency among all tumours. Other malignancies with a high prevalence of c-MYC pathway mutations were HNSC HPV- (85%), TCGT seminoma (72%), and high CN UCEC (70%). This finding indicated that the interaction of TLR and its downstream components in the c-MYC pathway played a role in the incidence and progression of these malignancies. Additionally, the String database's protein-protein interaction network established that the TLR family was intimately tied to not only the well-recognised NF-κB pathway but also the c-MYC pathway, especially MYC protein (Figure 7D).

### 3.10. Expression profiles of TLR family using single-cell RNA-Seq

According to Lee HO et al. [27], tumour and paired non-malignant colon samples of 23 Korean and 6 Belgian individuals diagnosed with colorectal cancer were brought into a scRNA-Seq method. The transcriptomes of 60,382 cells were effectively identified, and they were further classified into 36 clusters using a series of calculations and analyses (Figure S7A and S7B). In this investigation, we used the online procedure of Single Cell Expression Atlas to detect TLR gene expression, and some of the most significant results are shown in Figure S7 (whole results were displayed in Figure S8–S10). It was shown that TLR2 and TLR4 were predominantly expressed in myeloid cells, including SPP1+ B myeloid cells, proliferative myeloid cells, pro-inflammatory myeloid cells, and anti-inflammatory myeloid cells, as previously described. Additionally, TLR10 expression was localised in CD19 + CD20+ B cells. TLR7 and TLR9, on the other hand, did not aggregate in any cell clusters, despite the fact that TLR7 was uniformly low expressed and TLR9 was virtually completely absent.

In another study developed by Bassez A et al. [28], their scRNA-Seq dataset included 33,208 endothelial cells from three lung cancer patients. The website classified the mixture automatically into 21 clusters (Figure S11A), however, the data set did not include cell classification information. To our surprise, the link between TLR2 and TLR4 colocalization expression was still apparent in distinct subtypes of lung cancer endothelial cells (Figure S11C and S11D). Furthermore, the results above were similar to an HNCSS investigation using the scRNA-Seq technology, in which 164,276 cells from peripheral and intratumoral immune populations isolated from patients with HPV- and HPV + HNSCC were sequenced (Figure S12) [29].

### 3.11. Chromatin accessibility analysis of TLR family

We counted ATAC-Seq peaks in chromatin at TLR gene loci (from TLR1 to TLR10, displayed in Figure S13-S14) in several tumour cell lines to determine chromatin availability in pan-cancer. Consistent with the high level of mRNA expression, ATAC-Seq analysis revealed open regions with a dense population of peaks in all examined cell lines surrounding the TLR2 locus, near chr4:153700000 (Figure S15A). While the TLR4 gene had open sections in all cell lines, the density of peaks was lower than that of TLR2, and various cell lines had a range of TLR4 chromatin accessibility positions (Figure S15B). Near chr6:117900000, the NP-1 and NP-2 cell lines (2 human glioma cell lines) demonstrated more accessibility than the H1-hESC-1, H1-hESC-2, H9-hESC-1, H9-hESC-2 (4 human embryonic stem cell lines), MSiPS-1, and MSiPS-2 cell lines (2 human pluripotent stem cell lines). While LNCaP-1 and LNCaP2 (2 human prostate cancer cell lines) and H1437-1 and H1437-2 (2 human lung adenocarcinoma cell lines) demonstrated an open TLR4 gene region close to chr6:118050000. These data suggested that the differences in TLR4 expression between cancer types may be due to post-transcriptional alteration. There were no identifiable ATAC-Seq peaks for TLR7 and TLR9 in all cell lines, implying that chromatin accessibility inhibition was the source of low TLR7 and TLR9 mRNA expression.

## 4. Discussion

The pan-cancer analysis attempts to compare and contrast the genomic and cellular changes observed in different tumour forms in order to deduce a common mechanism shared by different cancer species. Other researchers have also integrated and studied 33 TCGA tumour samples to determine a variety of driving events associated with cancer formation and progression, such as mutations, chromosome fragmentation, and abnormal telomere maintenance, and etc [40].

TLRs, important components of the immune microenvironment, are widely recognised as single-pass membrane-spanning receptors equipped with an extracellular domain that particularly identify PAMPs and DAMPs. And, following activation, TLRs recruit adaptor proteins to the cytoplasm of immune cells, where they propagate antigen-induced signal transduction pathways, so initiating the anti-tumour immune response. However, high levels of TLR expression have been discovered in several tumour cells and tumour-associated macrophages, indicating that TLRs play a role in tumour growth as well. And the fascinating double-edged function of TLRs remains unknown, and its translational medicine application also needs additional research.

Many scientists, including Qiang Ju et al., have investigated the prognostic significance of many genes at the pan-cancer level. They investigated the expression patterns of molecules such as the immuno-related protein BRCA-1 and the anti-inflammatory and antioxidant-related transcription factor NRF2 in a range of malignancies at the genomic, protein expression, and clinical levels, as well as tumour immunologic analysis. A correlation study was performed between the degree of molecular expression and MMR gene mutation and DNA methylation at the genomic level. Survival analysis was done at the clinical level. In addition, tumour immunogenic correlation analysis was performed using correlation analysis of immune infiltration, immunological score, and immuno-checkpoint marker expression levels [41,42].

In our study, multi-omics analysis was used to elucidate the involvement of the TLR family in tumour growth and prognosis. We discovered that TLRs were significantly associated with tumour microenvironment, stem cell features, and clinical prognosis in pan-cancer and various single tumour data. As a result, TLR genes can be further investigated as a possible prognostic factor in clinical settings.

According to our findings, there is no consistent pattern of TLR expression across 33 distinct types of cancer, and the same TLR can be controlled differently depending on the kind of cancer (Figure 2), resulting in a favourable or negative prognosis (Figure 3). Here, we have concentrated on the function of TLR4, TLR7, and TLR9 in the cancer in this section.

TLR4 is the most frequently reported oncogenesis and tumour growth gene. It is located on chromosome 9 and encodes a 95680 Da protein with 839 amino acids. Compared with other molecules in the TLR family, TLR4 is relatively highly expressed at the transcriptome level in pan-cancer studies, close behind TLR2. While the majority of 33 forms of cancer exhibit decreased TLR4 expression in comparison to normal tissues, only a few cancers (such as GBM, KIRC, and STAD) exhibit the opposite trend. Additionally, there is a correlation between TLR4 and TLR7 (*R* = 0.66), TLR8 (*R* = 0.59), TLR1 (*R* = 0.56), TLR2 (*R* = 0.42), and TLR6 (*R* = 0.40), implying functional synergy. As demonstrated by a wide number of tests using heterodimers of TLR4 and TLR6 on the cell membrane. As for TME, in almost all malignancies except UVM, TLR4 is found to be positively linked with immune and stromal cell infiltration. Additionally, TLR4 expression is highest in C5 (Immunologically quiet), out of six immunological subtypes, and is also much greater than other TLRs in C5. As previously stated, C5 has the lowest lymphocyte count and the greatest macrophage count [14], which is dominated by M2-like macrophages, a kind of tumour-infiltrating myeloid cells. Likewise, TLR2 and TLR4 expression aggregated in a range of myeloid cells in colorectal tissue, implying that TLR4 activity is restricted to myeloid cells (macrophages, dendritic cells, monocytes etc.). Taken together, these findings suggest that therapeutic targeting of TLR4 in immunotherapy may be beneficial. TLR4 expression on CD103+ DC-like cells, for example, can boost CD8 + T cell infiltration in malignancies, making previously unresponsive cancers susceptible to checkpoint blockade therapy [43].

Concerning downstream signalling pathways, activation of TLR4/TLR6 heterodimers causes the generation of IL-1 in monocytes/macrophages *via* the NF-κB signalling pathway [44–47], and stimulation of monocytes induces the activation of p38 MAPK and ERK1/2 mostly *via* TLR2 but also partially *via* TLR4 [48]. Additionally, it is reported that β-catenin signalling can be triggered in colorectal cancer *via* a TLR4/P-PAK1 cascade [32].

As a result, TLR4 may aid in elucidating the underlying intracellular regulatory networks as well as the extracellular communication networks that govern the immune response to malignancies. DNAss and RNAss stemness scores estimated using the OCLR algorithm are both lowest in normal cells, increase in initial tumours, and are greatest in metastases [16,49]. Our findings indicate a substantial negative connection between TLR4 and RNAss in all TCGA tumour types, implying that TLR4 is not involved in biological processes active in cancer stem cells. TLR4 has a negative correlation with the majority of TCGA malignancies, but a positive connection with a small number of tumours, including ACC, CHOL, KIRP, LAML, PCPG, THCA, and THYM, indicating a possible relationship with specific epigenetic stemness traits in the cancer types mentioned. According to drug activity studies, TLR4 expression is associated with a lower number of chemotherapeutic agents than other TLRs. And the majority of the drugs described are either connected with inflammation and immunological response (e.g. progestin, histone deacetylase inhibitor, histone deacetylase inhibitor, HSP90 inhibitor, retinoid) or target TLR pathway downstream components (e.g. tyrosine kinase inhibitor). As TLR4 expression increases, cancer cells become more sensitive to medicines such as megestrol acetate, isotretinoin, entinostat, and okadaic acid. In these chemotherapeutic drugs, entinostat is most closely associated with TLR family members, and its sensitivity is also positively correlated with TLR1, TLR6, TLR7, TLR9, and TLR10, but negatively correlated with TLR8-AS1. Furthermore, the mechanism is based on the observation that inhibiting class I HDACs increases TLR signal transduction and cytokine generation in myeloid and T cells [50,51]. TLR4 is, however, associated with medication resistance to AT-13387, Acetalax, CUDC-305 By-Product, Lapatinib, and Ibrutinib. It has been demonstrated that Lapatinib and Ibrutinib, both of which are tyrosine kinase (BTK) inhibitors, partially dependent on the TLR family. The former inhibits the cytokine storm caused by TLR pathways [52], whereas the latter promotes apoptosis in CLL cells *via* TLR-7 and TLR-9 both *in vitro* and *in vivo* [53,54].

TLR7, which plays a critical role in innate and adaptive immunity, is typically found as homodimers on macrophages, B lymphocytes, and mast cells. The TLR7 gene is located on chromosomes X:12,867,072–12,890,361 and X:12,885,202–12,908,499. TLR7 expression is moderate in pan-cancer, lower than that of TLR4. It is primarily up-regulated throughout all TCGA malignancies, with exception of LUAD, LUSC, COAD, and READ. Apart from TLR4, co-expression occurs between TLR7 and TLR1 (*R* = 0.72), TLR8 and TLR4 (*R* = 0.69), TLR4 and TLR10 (*R* = 0.53), and TLR6 and TLR6 (*R* = 0.52). TLR7 is capable to recognise single strand RNAs (ssRNAs). Following ligand attachment, the stimulated TLR7 initiates downstream signal transduction by activating the transcription factors NF-κB and IRF7 [55–57]. Additionally, as our modification study revealed, the c-MYC pathway is a downstream mechanism for TLR3, TLR7, and TLR9 in a variety of malignancies [33–38].

In comparison to other TLRs, TLR7 expression is rather stable among the six immunological subtypes, being low in C1, C2, C3, and C4, and increasing from C5 to C6. Additionally, TLR7 and TLR8 are the only two members that exhibit a significant positive link with the level of immune and interstitial cell infiltration in all 33 cancer types, implying that TLR7 and TLR8 are associated with low tumour purity. Because immune infiltration inside tumour tissues indicates an adequate anti-tumour immune response, high TLR7 and TLR8 expression may predict a better patient prognosis, providing support for a wide range of immune therapies targeting TLR7 and TLR8 [58–61]. Rodell et al. recently demonstrated that a TLR7/8 agonist enhances polarisation of tumour-associated macrophages, hence enhancing anti-tumour therapy [60], but the detailed mechanism still needs further detection.

As for stemness features, TLR7 is often inversely linked with RNAss in the majority of malignancies. Additionally, TLR7 has been shown to alter the susceptibility of cancer cells to 30 medicines, with exception of Irofulven, a semi-synthetic analog of the fungal substance illudin S, which has a negative correlation, implying that an increase in TLR7 may result in Irofulven resistance. This tendency, however, is also observed in TLR9, and the chemical mechanism remains unknown.

TLR9's performance, which is likewise a critical component of innate and adaptive immunity, is particularly notable in the overall analysis results. This gene is located on chromosome 3 at positions 52,221,080–52,226,163 and 52,255,096–52,273,183. In contrast to TLR4 and TLR7, TLR9 forms both monomers and homodimers on the membrane and functions as a nucleotide-sensing TLR. Unmethylated cytidine-phosphate-guanosine (CpG) motifs (CpG ODNs), a TLR9 agonist, can stimulate antitumor immunity by activating the NF-κB pathway [5,62–64], and increase tumour cell death *via* cell cycle S phase arrest triggered by phosphorylated CHK2 [65–69]. Additionally, TLR9's identification of DNA factions assists in initiating T lymphocyte, B lymphocyte, and dendritic cell proliferation, activation, survival, and antibody production [68–71].

Notably, while TLR9 expression remains modest, it is up-regulated in all 33 tumours. The co-occurrence of TLR9 and TLRs is not as clear as that of other TLRs, and the most significant link is that of TLR6 co-expression (*R* = 0.32). Although there are considerable differences in TLR9 expression between the six immunological subtypes, no trend in TLR9 expression can be detected due to the expression level being too low. Additionally, the TME score analysis demonstrates that the association between TLR9 and many types of cancer exhibits a range of features, including positive correlation, negative correlation, and irrelevance, but the positive correlation is the predominant finding. TLR9 is negatively connected with RNAss in the majority of malignancies, but positively correlated with RNAss in CHOL, KIRC, and THYM, and has no correlation with RNAss in ACC, LIHC, PCPG, and UVM. However, there is no link between TLR9 and DNAss. It exhibits positive and negative relationships with 13 types of cancer but is unrelated to DNAss in the other seven types of cancer. The results above show that TLR9 may play a variety of roles in carcinogenesis and progression, which warrants additional investigation.

When it comes to drug action, all members of the TLR family influence the sensitivity of cells to medications in some way, but TLR9 has a particularly strong effect, as the sensitivity of up to 81 compounds is dependent on its expression level. Additionally, numerous studies have indicated that, in addition to its antitumor biological activity, TLR9 can enhance tumour cells' sensitivity to chemotherapy by increasing chemotherapy-induced apoptosis and decreasing tumour cell proliferation [72,73]. While up-regulation of TLR9 increases the sensitivity to the majority of medicines, only Irofulven, Kahalidof, Sonidogib, Trametimb, and Dasatinib are associated with drug resistance.

It is worth noting that Irofulven (MGI-114), a new cytotoxic anticancer drug with DNA damaging and MAPK activating properties, can also promote tumour cell apoptosis *via* ATM/CHK2 activation in the presence of BRCA1 [74–77]. Clearly, activated TLR9 and Irofulven share a common target, CHK2, and there is no indication that they have synergistic or antagonistic effects. However, it is intriguing to note that intracellular expression of c-MYC, a recognised proto-oncogene located downstream of TLR7 and TLR9, can modify tumour cell sensitivity to Irofulven [78]. This crosstalk may help explain drug resistance, but the precise mechanism remains unclear.

In a word, the pan-cancer research demonstrates that the TLR family expression pattern is strongly correlated with TME (both stromal and immune microenvironment), cancer stemness, and chemotherapy sensitivity, all of which vary among cancer types and subtypes. Additionally, TLR-activated signalling pathways result in the generation of cytokines, chemokines, and a variety of inducible molecules related to cell metabolism, indicating TLRs' significant function in tumour cells and the tumour microenvironment.

Although the effect of TLR on tumour prognosis has been documented in a significant number of tumour studies, this is the first study to integrate and analyse the TLR family of genes at the pan-cancer level, and to investigate their association with tumour formation. The findings indicate that TLR, as a possible prognostic marker, merits more investigation and discussion. However, the following unavoidable limitations remain: 1) This study analyzes mRNA-seq data from a single database, and the sample size is insufficient; 2) As a pure bioinformatics study, this study lacks experimental validation at the cellular or animal level; and 3) The data we collected are predominantly from Caucasians, and ethnic heterogeneity makes expansion difficult. As a result of this study's premise, we want to further investigate the role of TLR in carcinogenesis and development *via* cell research, animal investigations, and clinical trials, as well as to validate TLR's utility as a potential clinical biomarker.

## Conclusion

In our research, we investigated the TLR family's functions at the transcriptomic, genomic, and proteomic levels in 33 TCGA malignancies and also validated them using single-cell data and chromosome accessibility studies. The results indicate that the TLR gene plays a role in tumour growth and has an effect on tumour cells' sensitivity to chemotherapy. As a consequence, we believe that TLRs, particularly TLR4, TLR7, and TLR9, may be useful as predictive biomarkers.

## Supplementary Material

Supplemental Material

## Data Availability

All data was processed by Perl (Practical Extraction and Report Language, https://www.perl.org), R software version 3.6.1 (http://www.r-project.org; Institute for Statistics and Mathematics, Vienna, Austria) and packages acquired from Bioconductor (http://www.bioconductor.org/packages/release/bioc/html/impute.html). Our source data and code are uploaded as supplementary files.

## Data availability statement

The datasets generated and/or analysed during the current study are available in the TCGA program (https://portal.gdc.cancer.gov).

## References

[1] Newton K, Dixit VM. Signaling in innate immunity and inflammation. Cold Spring Harb Perspect Biol. 2012;4(3). 10.1101/cshperspect.a006049 PMC3282411 22296764

[2] Celhar T, Magalhaes R, Fairhurst AM. TLR7 and TLR9 in SLE: when sensing self goes wrong. Immunol Res. 2012;53(1–3):58–77. 22434514 10.1007/s12026-012-8270-1

[3] Kawai T, Akira S. The role of pattern-recognition receptors in innate immunity: update on toll-like receptors. Nat Immunol. 2010;11(5):373–384. 20404851 10.1038/ni.1863

[4] Tartey S, Takeuchi O. Pathogen recognition and toll-like receptor targeted therapeutics in innate immune cells. Int Rev Immunol. 2017;36(2):57–73. 28060562 10.1080/08830185.2016.1261318

[5] Janeway CA Jr, Medzhitov R. Innate immune recognition. Annu Rev Immunol. 2002;20:197–216. 11861602 10.1146/annurev.immunol.20.083001.084359

[6] Shime H, Matsumoto M, Oshiumi H, et al. Toll-like receptor 3 signaling converts tumor-supporting myeloid cells to tumoricidal effectors. Proc Natl Acad Sci USA. 2012;109(6):2066–2071. 22308357 10.1073/pnas.1113099109 PMC3277567

[7] Zhang Y, Yu G, Chu H, et al. Macrophage-associated PGK1 phosphorylation promotes aerobic glycolysis and tumorigenesis. Mol Cell. 2018;71(2):201–215 e7. 30029001 10.1016/j.molcel.2018.06.023

[8] Kauppila JH, Selander KS. Toll-like receptors in esophageal cancer. Front Immunol. 2014;5(200):200. 24847326 10.3389/fimmu.2014.00200 PMC4019875

[9] Sheyhidin I, Nabi G, Hasim A, et al. Overexpression of TLR3, TLR4, TLR7 and TLR9 in esophageal squamous cell carcinoma. WJG. 2011;17(32):3745–3751. 21990957 10.3748/wjg.v17.i32.3745 PMC3181461

[10] Chen R, Alvero AB, Silasi DA, et al. Inflammation, cancer and chemoresistance: taking advantage of the toll-like receptor signaling pathway. Am J Reprod Immunol. 2007;57(2):93–107. 17217363 10.1111/j.1600-0897.2006.00441.x

[11] Dajon M, Iribarren K, Cremer I. Toll-like receptor stimulation in cancer: a pro- and anti-tumor double-edged sword. Immunobiology. 2017;222(1):89–100. 27349597 10.1016/j.imbio.2016.06.009

[12] Sanchez-Vega F, Mina M, Armenia J, et al. Oncogenic signaling pathways in the cancer genome atlas. Cell. 2018;173(2):321–37.e10. 29625050 10.1016/j.cell.2018.03.035 PMC6070353

[13] Corces MR, Granja JM, Shams S, et al. The chromatin accessibility landscape of primary human cancers. Science. 2018;362(6413):eaav1898. 30361341 10.1126/science.aav1898 PMC6408149

[14] Thorsson V, Gibbs DL, Brown SD, et al. The immune landscape of cancer. Immunity. 2018;48(4):812–30 e14. 29628290 10.1016/j.immuni.2018.03.023 PMC5982584

[15] Yoshihara K, Shahmoradgoli M, Martinez E, et al. Inferring tumour purity and stromal and immune cell admixture from expression data. Nat Commun. 2013;4:2612. 24113773 10.1038/ncomms3612 PMC3826632

[16] Malta TM, Sokolov A, Gentles AJ, et al. Machine learning identifies stemness features associated with oncogenic dedifferentiation. Cell. 2018;173(2):338–54 e15. 29625051 10.1016/j.cell.2018.03.034 PMC5902191

[17] Shankavaram UT, Varma S, Kane D, et al. CellMiner: a relational database and query tool for the NCI-60 cancer cell lines. BMC Genomics. 2009;10:277. 19549304 10.1186/1471-2164-10-277 PMC2709662

[18] Reinhold WC, Sunshine M, Liu H, et al. CellMiner: a web-based suite of genomic and pharmacologic tools to explore transcript and drug patterns in the NCI-60 cell line set. Cancer Res. 2012;72(14):3499–3511. 22802077 10.1158/0008-5472.CAN-12-1370 PMC3399763

[19] RM S, CH L, AN S, et al. Tumor mutational load predicts survival after immunotherapy across multiple cancer types. Nat Genet. 2019;51(2):202–206. 30643254 10.1038/s41588-018-0312-8 PMC6365097

[20] MS L, P S, P P, et al. Mutational heterogeneity in cancer and the search for new cancer-associated genes. Nature. 2013;499(7457):214–218. 23770567 10.1038/nature12213 PMC3919509

[21] Cerami E, Gao J, Dogrusoz U, et al. The cBio cancer genomics portal: an open platform for exploring multidimensional cancer genomics data. Cancer Discov. 2012;2(5):401–404. 22588877 10.1158/2159-8290.CD-12-0095 PMC3956037

[22] Thul PJ, Lindskog C. The human protein atlas: a spatial map of the human proteome. Protein Sci. 2018;27(1):233–244. 28940711 10.1002/pro.3307 PMC5734309

[23] Vasaikar SV, Straub P, Wang J, et al. LinkedOmics: analyzing multi-omics data within and across 32 cancer types. Nucleic Acids Res. 2018;46(D1):D956–D63. 29136207 10.1093/nar/gkx1090 PMC5753188

[24] Ashburner M, Ball CA, Blake JA, et al. Gene ontology: tool for the unification of biology. The gene ontology consortium. Nat Genet. 2000;25(1):25–29. 10802651 10.1038/75556 PMC3037419

[25] Ogata H, Goto S, Sato K, et al. KEGG: kyoto encyclopedia of genes and genomes. Nucleic Acids Res. 1999;27(1):29–34. 9847135 10.1093/nar/27.1.29 PMC148090

[26] Szklarczyk D, Gable AL, Lyon D, et al. STRING v11: protein-protein association networks with increased coverage, supporting functional discovery in genome-wide experimental datasets. Nucleic Acids Res. 2019;47(D1):D607–D13. 30476243 10.1093/nar/gky1131 PMC6323986

[27] Lee HO, Hong Y, Etlioglu HE, et al. Lineage-dependent gene expression programs influence the immune landscape of colorectal cancer. Nat Genet. 2020;52(6):594–603. 32451460 10.1038/s41588-020-0636-z

[28] Lambrechts D, Wauters E, Boeckx B, et al. Phenotype molding of stromal cells in the lung tumor microenvironment. Nat Med. 2018;24(8):1277–1289. 29988129 10.1038/s41591-018-0096-5

[29] Cillo AR, Kurten CHL, Tabib T, et al. Immune landscape of viral- and carcinogen-driven head and neck cancer. Immunity. 2020;52(1):183–199 e9. 31924475 10.1016/j.immuni.2019.11.014 PMC7201194

[30] Papatheodorou I, Moreno P, Manning J, et al. Expression atlas update: from tissues to single cells. Nucleic Acids Res. 2020;48(D1):D77–D83. 31665515 10.1093/nar/gkz947 PMC7145605

[31] Buenrostro JD, Giresi PG, Zaba LC, et al. Transposition of native chromatin for fast and sensitive epigenomic profiling of open chromatin, DNA-binding proteins and nucleosome position. Nat Methods. 2013;10(12):1213–1218. 24097267 10.1038/nmeth.2688 PMC3959825

[32] Chen Y, Peng Y, Yu J, et al. Invasive fusobacterium nucleatum activates beta-catenin signaling in colorectal cancer via a TLR4/P-PAK1 cascade. Oncotarget. 2017;8(19):31802–31814. 28423670 10.18632/oncotarget.15992 PMC5458249

[33] Feist M, Kemper J, Taruttis F, et al. Synergy of interleukin 10 and toll-like receptor 9 signalling in B cell proliferation: Implications for lymphoma pathogenesis. Int J Cancer. 2017;140(5):1147–1158. 27668411 10.1002/ijc.30444

[34] Lin LL, Huang CC, Wu CL, et al. Downregulation of c-Myc is involved in TLR3-mediated tumor death of neuroblastoma xenografts. Lab Invest. 2016;96(7):719–730. 27183205 10.1038/labinvest.2016.57

[35] Liu H, Schwartz MJ, Hwang DH, et al. Tumour growth inhibition by an imidazoquinoline is associated with c-Myc down-regulation in urothelial cell carcinoma. BJU Int. 2008;101(7):894–901. 18241249 10.1111/j.1464-410X.2008.07459.x

[36] Matijevic Glavan T, Cipak Gasparovic A, Verillaud B, et al. Toll-like receptor 3 stimulation triggers metabolic reprogramming in pharyngeal cancer cell line through myc, MAPK, and HIF. Mol Carcinog. 2017;56(4):1214–1226. 27805282 10.1002/mc.22584

[37] Ochi A, Graffeo CS, Zambirinis CP, et al. Toll-like receptor 7 regulates pancreatic carcinogenesis in mice and humans. J Clin Invest. 2012;122(11):4118–4129. 23023703 10.1172/JCI63606 PMC3484447

[38] Pries R, Hogrefe L, Xie L, et al. Induction of c-Myc-dependent cell proliferation through toll-like receptor 3 in head and neck cancer. Int J Mol Med. 2008;21(2):209–215. 18204787

[39] Shi Y, Tomic J, Wen F, et al. Aberrant O-GlcNAcylation characterizes chronic lymphocytic leukemia. Leukemia. 2010;24(9):1588–1598. 20668475 10.1038/leu.2010.152 PMC4361888

[40] Consortium I-C. Pan-cancer analysis of whole genomes. Nature. 2020;578(7793):82–93. 32025007 10.1038/s41586-020-1969-6 PMC7025898

[41] Ju Q, Li X, Zhang H, et al. NFE2L2 is a potential prognostic biomarker and is correlated with immune infiltration in brain lower grade glioma: a pan-cancer analysis. Oxid Med Cell Longev. 2020;2020:3580719. 33101586 10.1155/2020/3580719 PMC7569466

[42] Ju Q, Li XM, Zhang H, et al. BRCA1-associated protein is a potential prognostic biomarker and is correlated with immune infiltration in liver hepatocellular carcinoma: a pan-cancer analysis. Front Mol Biosci. 2020;7:573619. 33240929 10.3389/fmolb.2020.573619 PMC7667264

[43] Pfirschke C, Engblom C, Rickelt S, et al. Immunogenic chemotherapy sensitizes tumors to checkpoint blockade therapy. Immunity. 2016;44(2):343–354. 26872698 10.1016/j.immuni.2015.11.024 PMC4758865

[44] Estruch M, Bancells C, Beloki L, et al. CD14 and TLR4 mediate cytokine release promoted by electronegative LDL in monocytes. Atherosclerosis. 2013;229(2):356–362. 23880187 10.1016/j.atherosclerosis.2013.05.011

[45] Arbour NC, Lorenz E, Schutte BC, et al. TLR4 mutations are associated with endotoxin hyporesponsiveness in humans. Nat Genet. 2000;25(2):187–191. 10835634 10.1038/76048

[46] Medzhitov R, Preston-Hurlburt P, Janeway CA Jr. A human homologue of the drosophila toll protein signals activation of adaptive immunity. Nature. 1997;388(6640):394–397. 9237759 10.1038/41131

[47] Tatematsu M, Yoshida R, Morioka Y, et al. Raftlin controls lipopolysaccharide-induced TLR4 internalization and TICAM-1 signaling in a cell type-specific manner. J Immunol. 2016;196(9):3865–3876. 27022195 10.4049/jimmunol.1501734

[48] Jung SB, Yang CS, Lee JS, et al. The mycobacterial 38-kilodalton glycolipoprotein antigen activates the mitogen-activated protein kinase pathway and release of proinflammatory cytokines through toll-like receptors 2 and 4 in human monocytes. Infect Immun. 2006;74(5):2686–2696. 16622205 10.1128/IAI.74.5.2686-2696.2006 PMC1459749

[49] Sokolov A, Paull EO, Stuart JM. One-class detection of cell states in tumor subtypes. Pac Symp Biocomput. 2016;21:405–416. 26776204 PMC4856035

[50] Shakespear MR, Halili MA, Irvine KM, et al. Histone deacetylases as regulators of inflammation and immunity. Trends Immunol. 2011;32(7):335–343. 21570914 10.1016/j.it.2011.04.001

[51] Klampfer L, Huang J, Swaby LA, et al. Requirement of histone deacetylase activity for signaling by STAT1. J Biol Chem. 2004;279(29):30358–30368. 15123634 10.1074/jbc.M401359200

[52] Zhao S, Gao N, Qi H, et al. Suppressive effects of sunitinib on a TLR activation-induced cytokine storm. Eur J Pharmacol. 2019;854:347–353. 31039345 10.1016/j.ejphar.2019.04.045

[53] Dadashian EL, McAuley EM, Liu D, et al. TLR signaling is activated in lymph Node-Resident CLL cells and is only partially inhibited by ibrutinib. Cancer Res. 2019;79(2):360–371. 30498085 10.1158/0008-5472.CAN-18-0781 PMC6342512

[54] Chakraborty R, Kapoor P, Ansell SM, et al. Ibrutinib for the treatment of waldenstrom macroglobulinemia. Expert Rev Hematol. 2015;8(5):569–579. 26138997 10.1586/17474086.2015.1061427

[55] Davenne T, Bridgeman A, Rigby RE, et al. Deoxyguanosine is a TLR7 agonist. Eur J Immunol. 2020;50(1):56–62. 31608988 10.1002/eji.201948151 PMC6972671

[56] Lee J, Chuang TH, Redecke V, et al. Molecular basis for the immunostimulatory activity of guanine nucleoside analogs: activation of toll-like receptor 7. Proc Natl Acad Sci USA. 2003;100(11):6646–6651. 12738885 10.1073/pnas.0631696100 PMC164501

[57] Zhang Z, Ohto U, Shibata T, et al. Structural analysis reveals that toll-like receptor 7 is a dual receptor for guanosine and single-stranded RNA. Immunity. 2016;45(4):737–748. 27742543 10.1016/j.immuni.2016.09.011

[58] Adams S, Kozhaya L, Martiniuk F, et al. Topical TLR7 agonist imiquimod can induce immune-mediated rejection of skin metastases in patients with breast cancer. Clin Cancer Res. 2012;18(24):6748–6757. 22767669 10.1158/1078-0432.CCR-12-1149 PMC3580198

[59] Schon MP, Schon M. TLR7 and TLR8 as targets in cancer therapy. Oncogene. 2008;27(2):190–199. 18176600 10.1038/sj.onc.1210913

[60] Rodell CB, Arlauckas SP, Cuccarese MF, et al. TLR7/8-agonist-loaded nanoparticles promote the polarization of tumour-associated macrophages to enhance cancer immunotherapy. Nat Biomed Eng. 2018;2(8):578–588. 31015631 10.1038/s41551-018-0236-8 PMC6192054

[61] Michaelis KA, Norgard MA, Zhu X, et al. The TLR7/8 agonist R848 remodels tumor and host responses to promote survival in pancreatic cancer. Nat Commun. 2019;10(1):4682. 31615993 10.1038/s41467-019-12657-w PMC6794326

[62] Doyle SL, Jefferies CA, Feighery C, et al. Signaling by toll-like receptors 8 and 9 requires Bruton's tyrosine kinase. J Biol Chem. 2007;282(51):36953–36960. 17932028 10.1074/jbc.M707682200

[63] Takeshita F, Leifer CA, Gursel I, et al. Cutting edge: role of toll-like receptor 9 in CpG DNA-induced activation of human cells. J Immunol. 2001;167(7):3555–3558. 11564765 10.4049/jimmunol.167.7.3555

[64] Akira S, Uematsu S, Takeuchi O. Pathogen recognition and innate immunity. Cell. 2006;124(4):783–801. 16497588 10.1016/j.cell.2006.02.015

[65] Chen W, Liu X, Qiao T, et al. Impact of CHK2-small interfering RNA on CpG ODN7909-enhanced radiosensitivity in lung cancer A549 cells. Onco Targets Ther. 2012;5:425–431. 23233807 10.2147/OTT.S38240 PMC3518288

[66] Kumagai Y, Takeuchi O, Akira S. TLR9 as a key receptor for the recognition of DNA. Adv Drug Deliv Rev. 2008;60(7):795–804. 18262306 10.1016/j.addr.2007.12.004

[67] Brignole C, Marimpietri D, Di Paolo D, et al. Therapeutic targeting of TLR9 inhibits cell growth and induces apoptosis in neuroblastoma. Cancer Res. 2010;70(23):9816–9826. 20935225 10.1158/0008-5472.CAN-10-1251

[68] Okamoto M, Sato M. Toll-like receptor signaling in anti-cancer immunity. J Med Invest. 2003;50(1-2):9–24. 12630564

[69] Shi R, Hong L, Wu D, et al. Enhanced immune response to gastric cancer specific antigen peptide by coencapsulation with CpG oligodeoxynucleotides in nanoemulsion. Cancer Biol Ther. 2005;4(2):218–224. 15753659 10.4161/cbt.4.2.1472

[70] Li FJ, Schreeder DM, Li R, et al. FCRL3 promotes TLR9-induced B-cell activation and suppresses plasma cell differentiation. Eur J Immunol. 2013;43(11):2980–2992. 23857366 10.1002/eji.201243068 PMC3838486

[71] Zheng L, Asprodites N, Keene AH, et al. TLR9 engagement on CD4 T lymphocytes represses gamma-radiation-induced apoptosis through activation of checkpoint kinase response elements. Blood. 2008;111(5):2704–2713. 18086870 10.1182/blood-2007-07-104141 PMC2254540

[72] Wang H, Rayburn ER, Wang W, et al. Chemotherapy and chemosensitization of non-small cell lung cancer with a novel immunomodulatory oligonucleotide targeting toll-like receptor 9. Mol Cancer Ther. 2006;5(6):1585–1592. 16818518 10.1158/1535-7163.MCT-06-0094

[73] Rayburn ER, Wang W, Zhang R, et al. Experimental therapy for colon cancer: anti-cancer effects of TLR9 agonism, combination with other therapeutic modalities, and dependence upon p53. Int J Oncol. 2007;30(6):1511–1519. 17487373

[74] Wang J, Wiltshire T, Wang Y, et al. ATM-dependent CHK2 activation induced by anticancer agent, irofulven. J Biol Chem. 2004;279(38):39584–39592. 15269203 10.1074/jbc.M400015200

[75] Wiltshire TD. DNA damage response activated by anti-cancer agent. Irofulven. 2007;3300923:236.

[76] Woynarowska BA, Woynarowski JM. Preferential targeting of apoptosis in tumor versus normal cells. Biochim Biophys Acta. 2002;1587(2–3):309–317. 12084473 10.1016/s0925-4439(02)00094-7

[77] Sakthivel KM, Hariharan S. Regulatory players of DNA damage repair mechanisms: role in cancer chemoresistance. Biomed Pharmacother. 2017;93:1238–1245. 28738540 10.1016/j.biopha.2017.07.035

[78] Kelner MJ, McMorris TC, Tsigelny IS, et al. Role of c-myc expression in cellular sensitivity of irofulven. Cancer Res. 2004;64:131.

