# Supplementary material for: The role of toll-like receptors (TLRs) in pan-cancer
Source: Ann Med. 2022 Jul 8;54(1):1918–37. doi: 10.1080/07853890.2022.2095664 (PMC9272932; doi:10.1080/07853890.2022.2095664)
Supplement: Supplemental Material [file IANN_A_2095664_SM1580.docx]

## Supplementary Materials

##
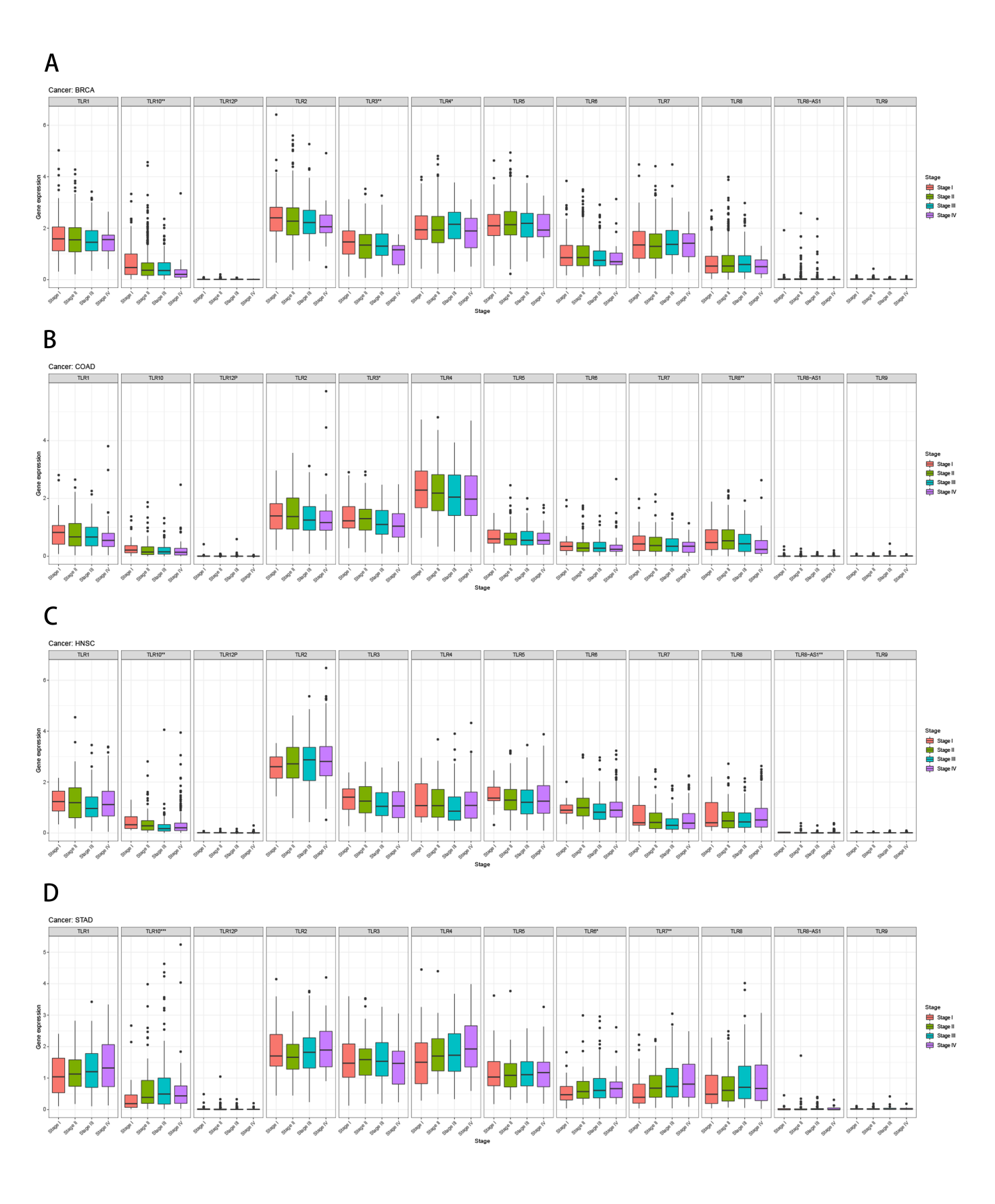


## Figure S1 Clinical correlation analysis of TLR gene in 4 single tumor data. From (A) to (D) were BRCA, COAD, HNSC and STAD.


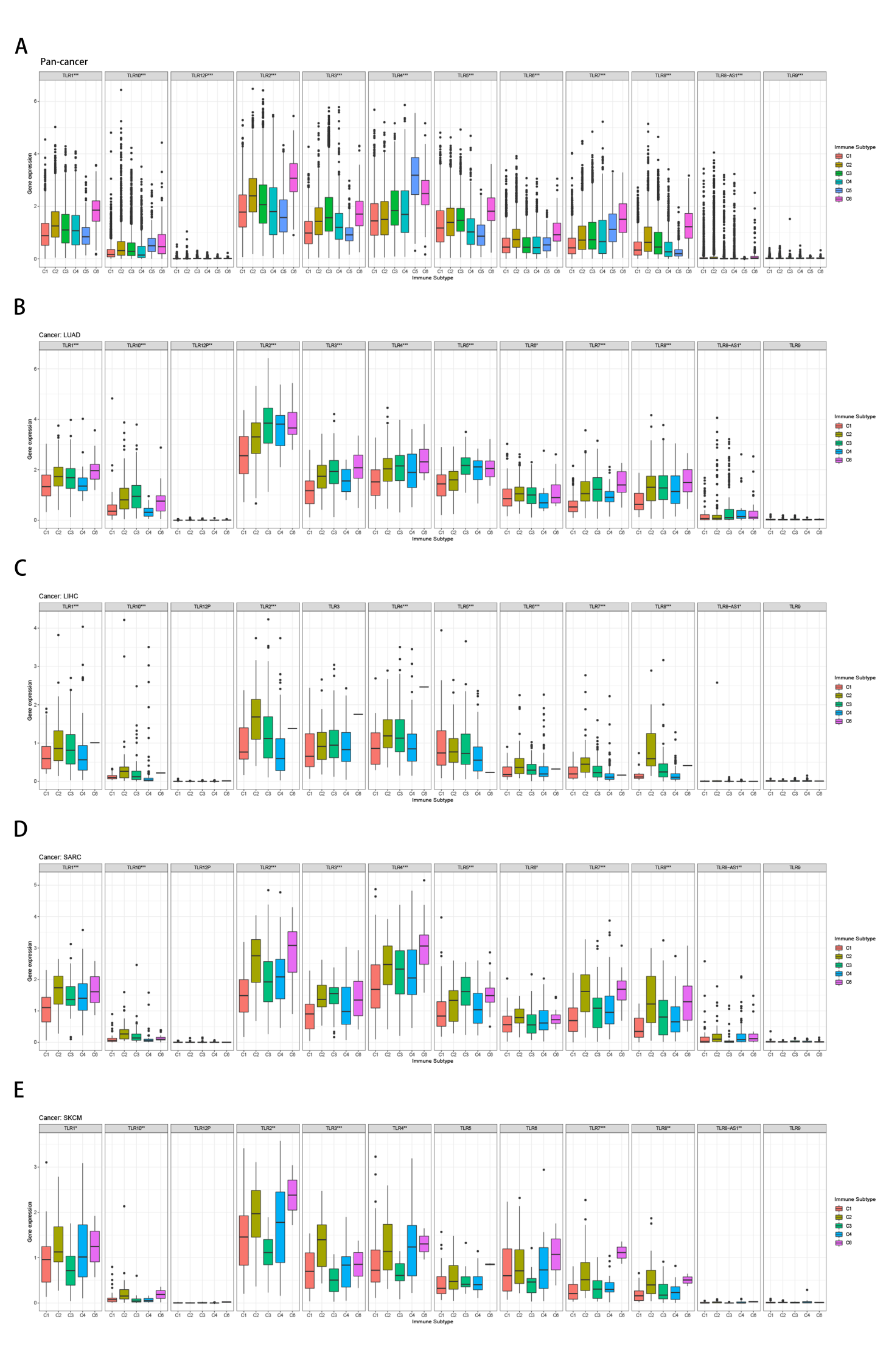


## Figure S2 Immune subtype analysis in pan-caner (A) and single tumor data. From (B) to (E) were LUAD, LIHC, SARC, SKCM.


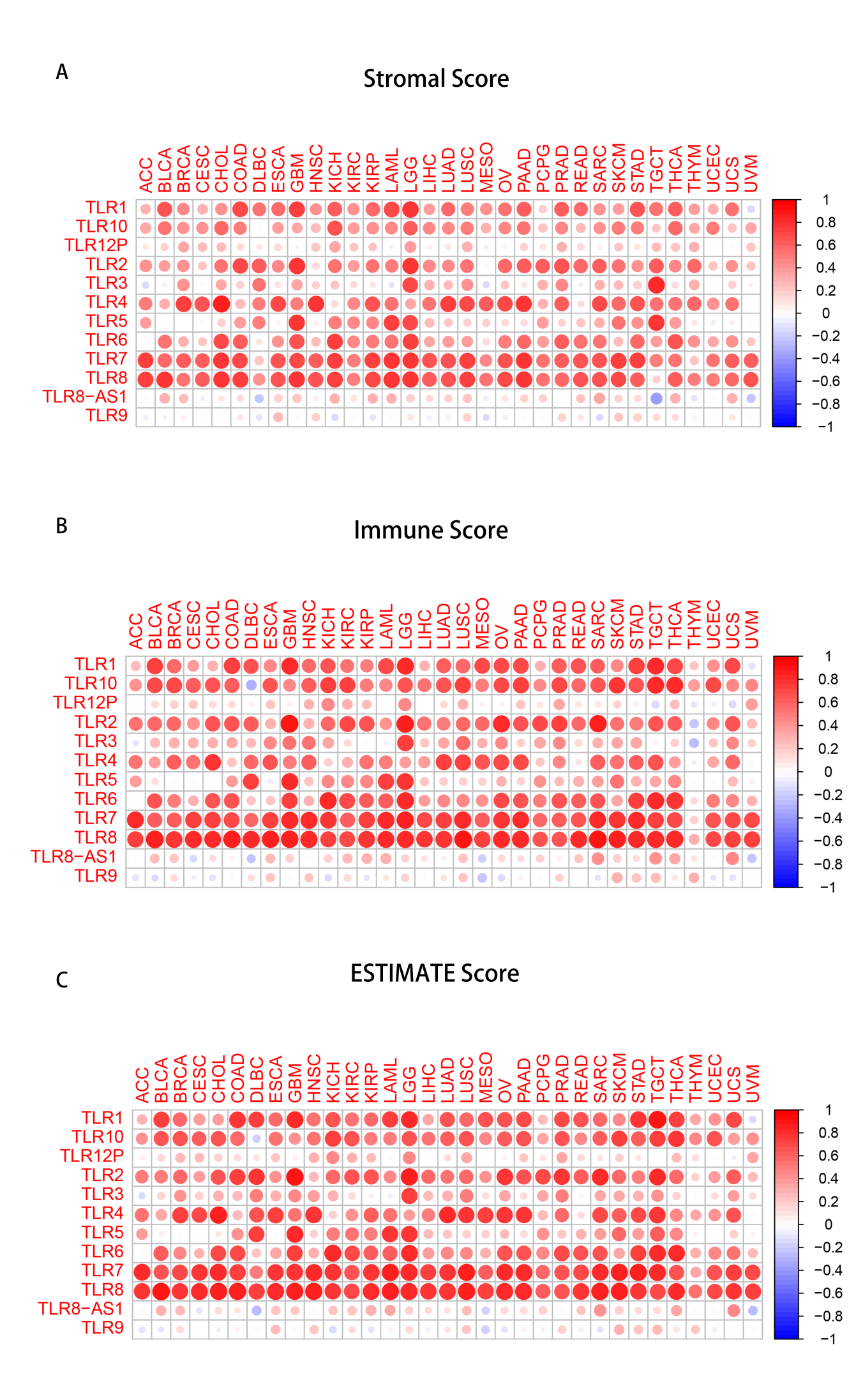


## Figure S3 Tumor stemness correlation of TLR family in pan-cancer. The result of correlation analysis between TLR family and stromal score (A), immune score (B) and estimate score (C): the red and blue dots represent positive and negative correlations respectively, and the density reflects the correlation coefficient.

##
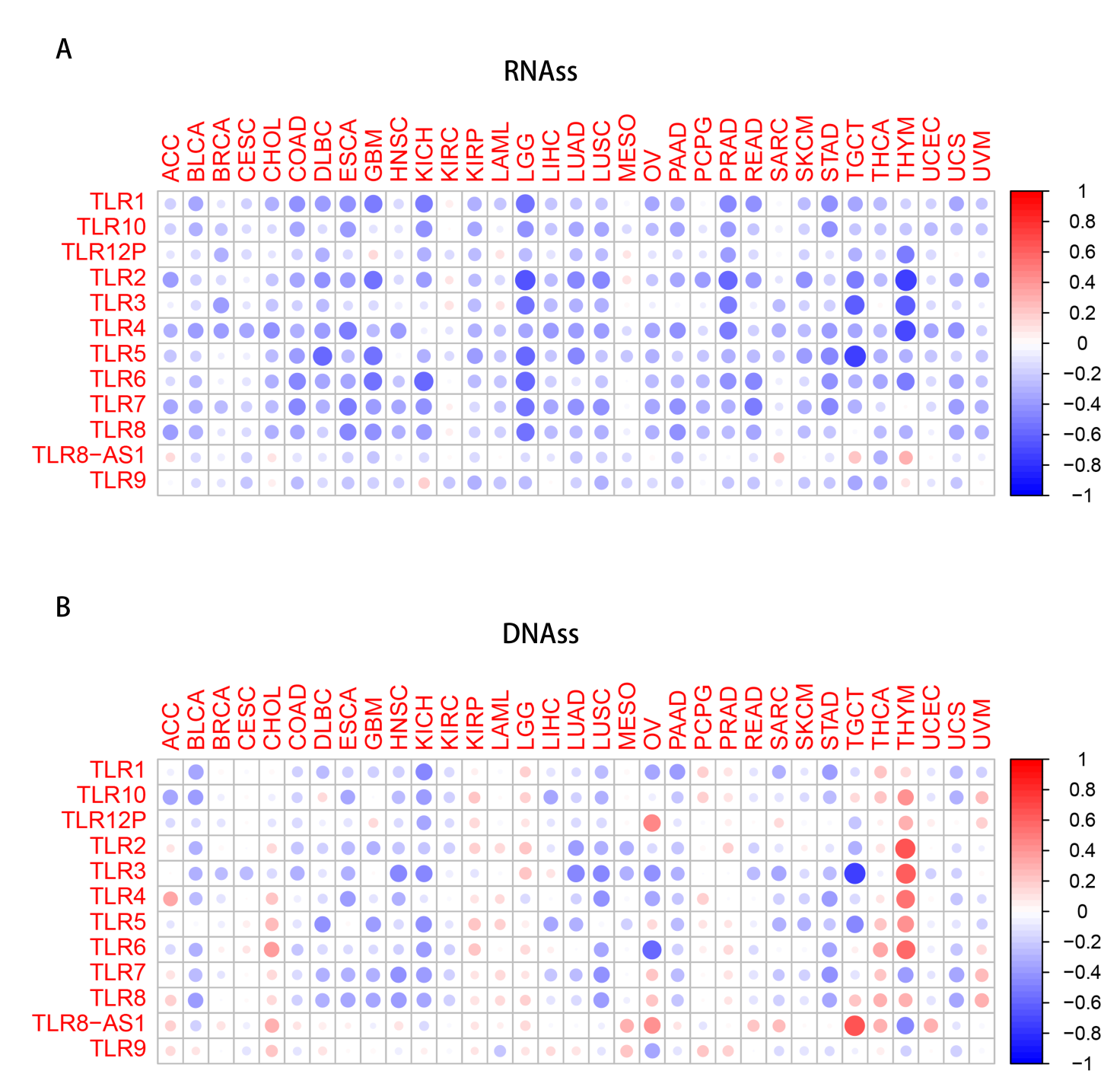


## Figure S4 The correlation analysis between expression of TLR genes and stemness score, including RNAss (A) and DNAss (B).


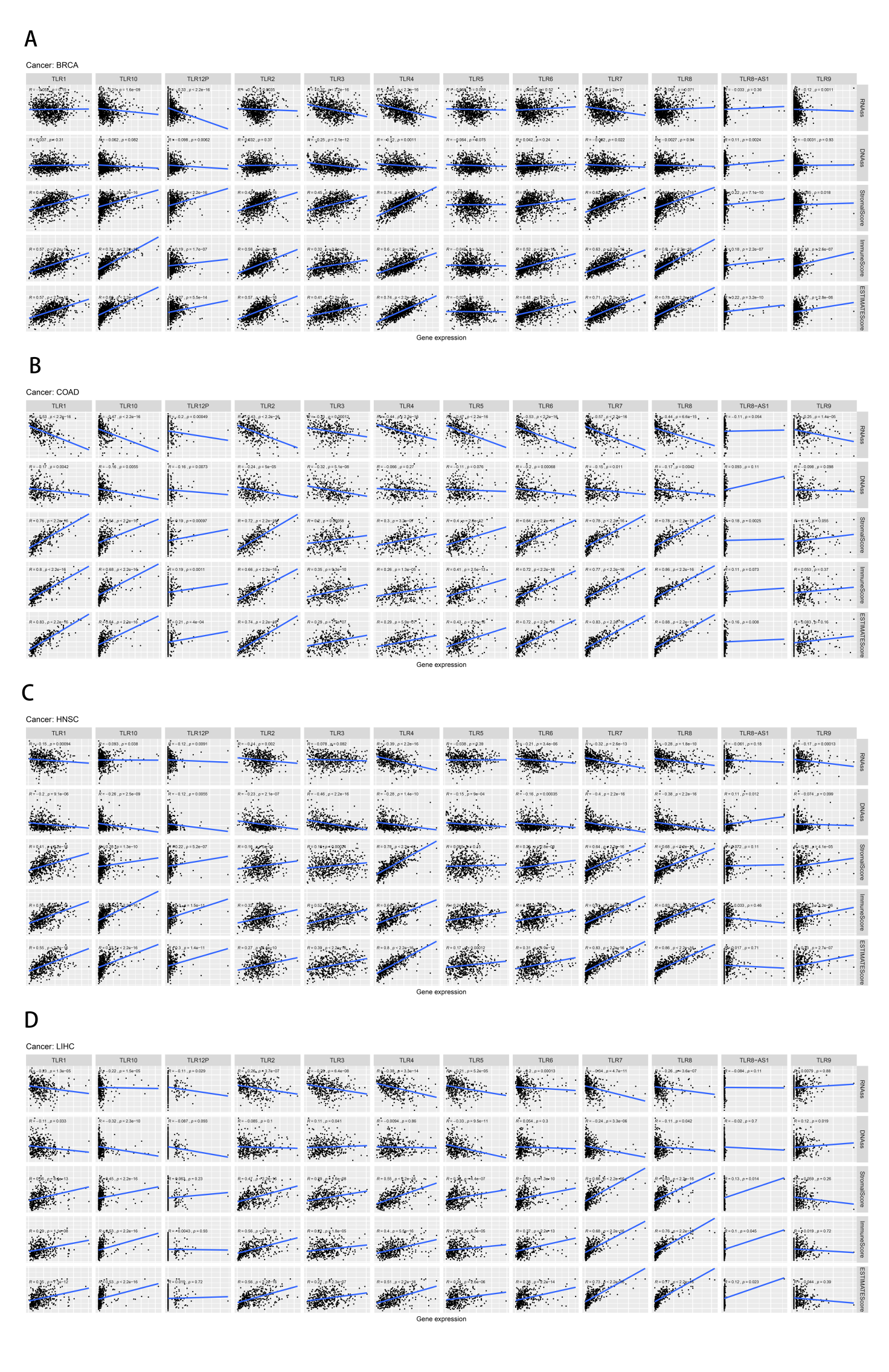


## Figure S5 The tumor microenvironment and tumor stemness correlation analysis in 4 single cancer data. From (A) to (D) were BRCA, COAD, HNSC and LINC.


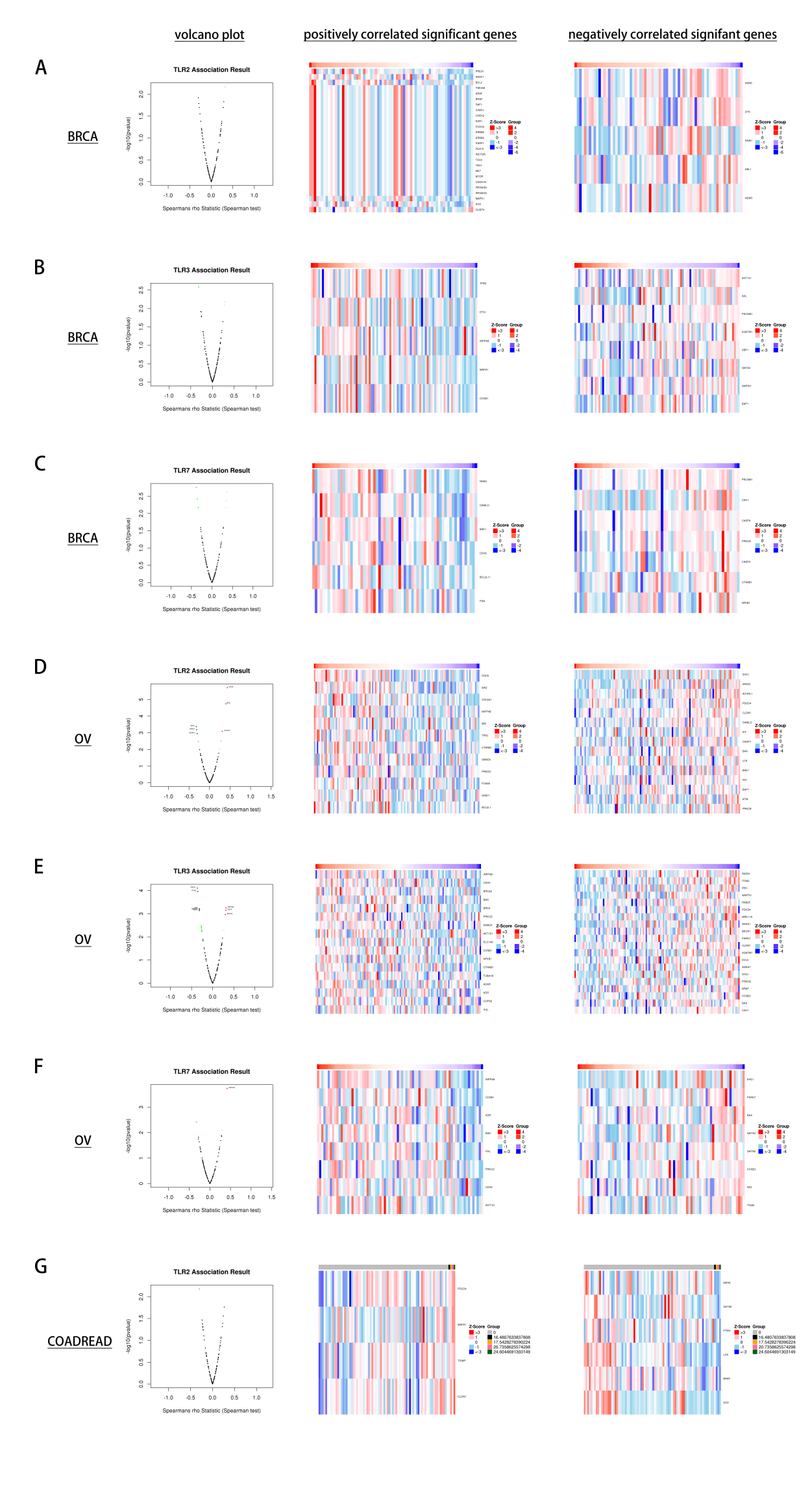


## Figure S6 Positive and negative correlation between the mRNA level of TLR2, TLR3, TLR7 and protein detected by reverse phase protein microarray (RPPA): the density of color indicates the Z-score in the co-expression relationship.

##
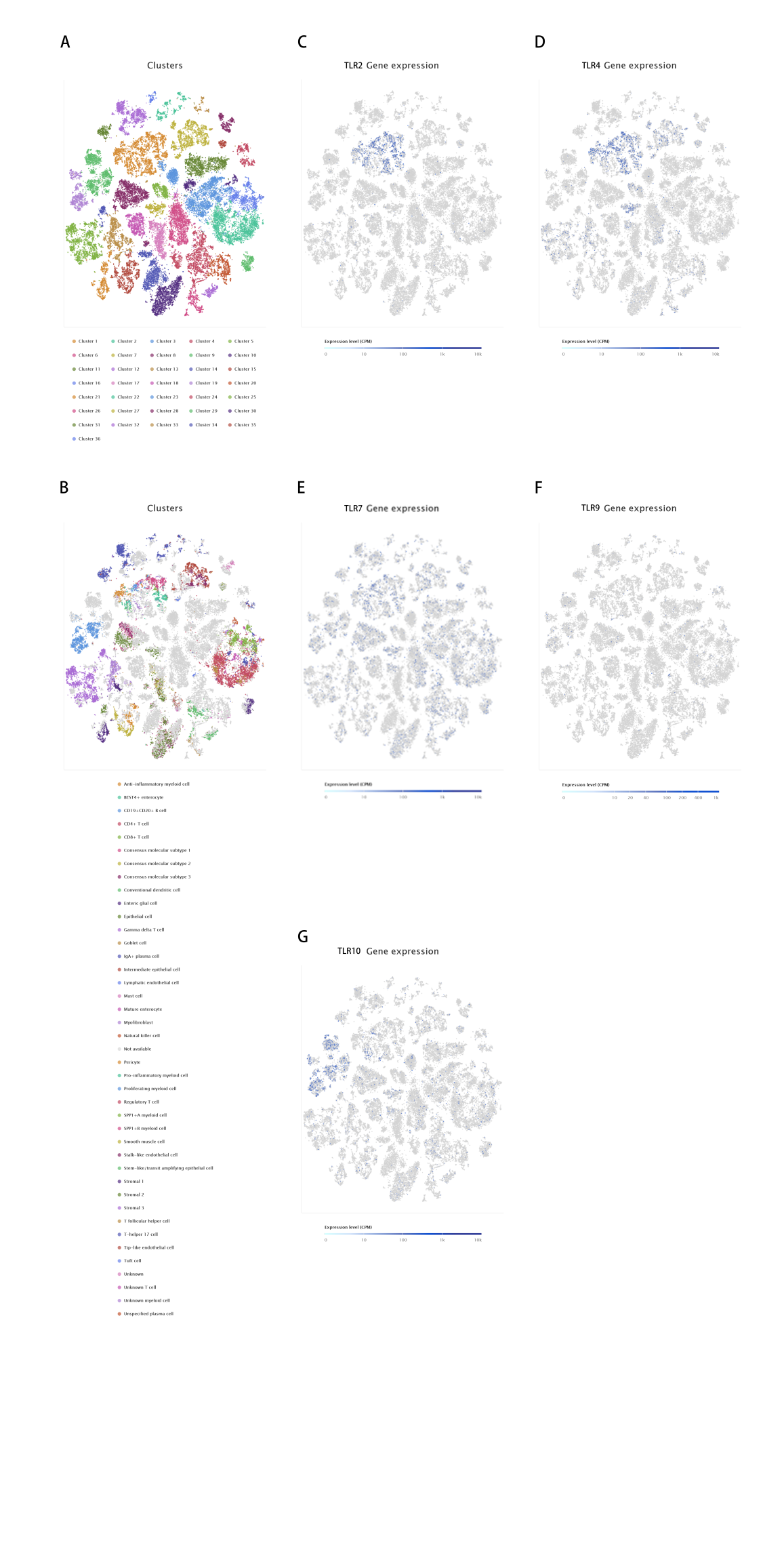


## Figure S7 Single cell sequencing of human colorectal tumors and adjacent tissues.

## Mixture of cells were divided into 36 clusters.

## Inferred cell types annotated by authors.

## (C -G) Expression of TLR2, TLR4, TLR7, TLR9 and TLR10 at the single cell level.

##
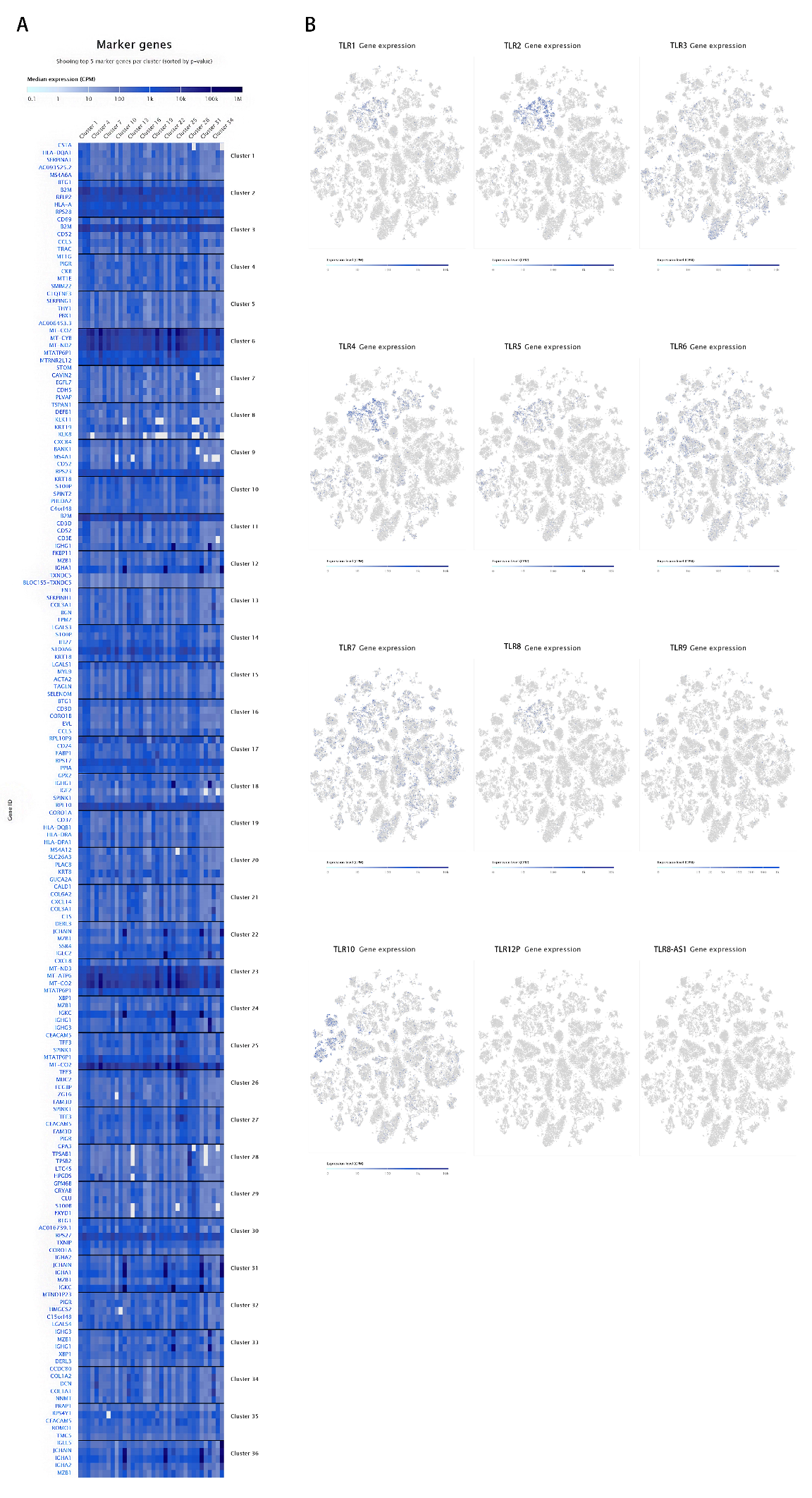


## Figure S8 Single cell sequencing of human colorectal tumors and adjacent tissues.

## Marker genes of each cluster.

## (B to M) Expression of TLR1, TLR2, TLR3, TLR4, TLR5, TLR6, TLR7, TLR8, TLR9, TLR10, TLR8-AS1, TLR12P.


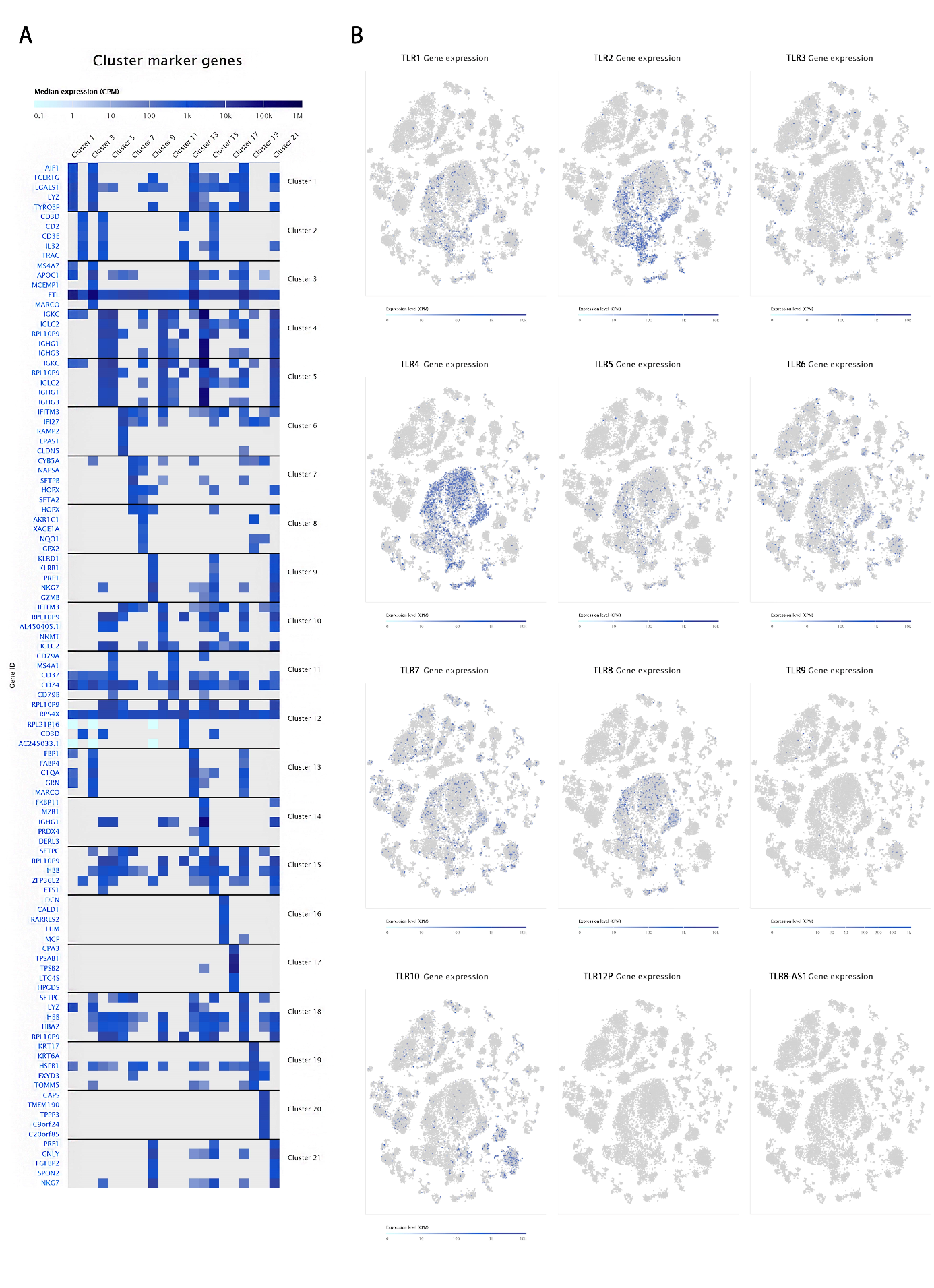


## Figure S9 Single cell sequencing of lung endothelial cells and tumor endothelial cells form NSCLC patients.

## Marker genes of each cluster.

## (B-M) Expression of TLR1, TLR2, TLR3, TLR4, TLR5, TLR6, TLR7, TLR8, TLR9, TLR10, TLR8-AS1, TLR12P.

##
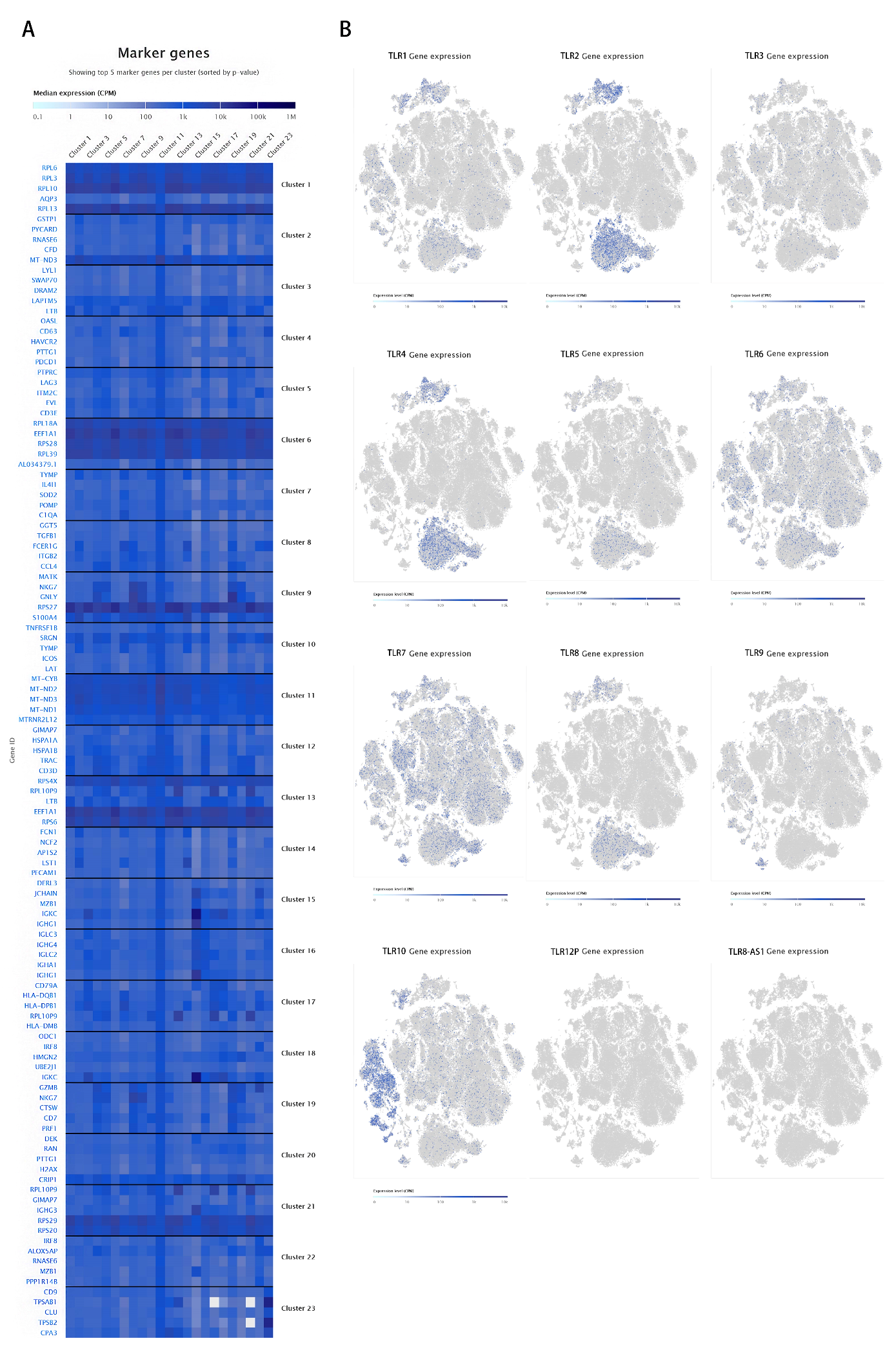


## Figure S10 Single cell sequencing of human head and neck squamous carcinoma tissues and paired normal tissues.

## Marker genes of each cluster.

## (B-M) Expression of TLR1, TLR2, TLR3, TLR4, TLR5, TLR6, TLR7, TLR8, TLR9, TLR10, TLR8-AS1, TLR12P.

##
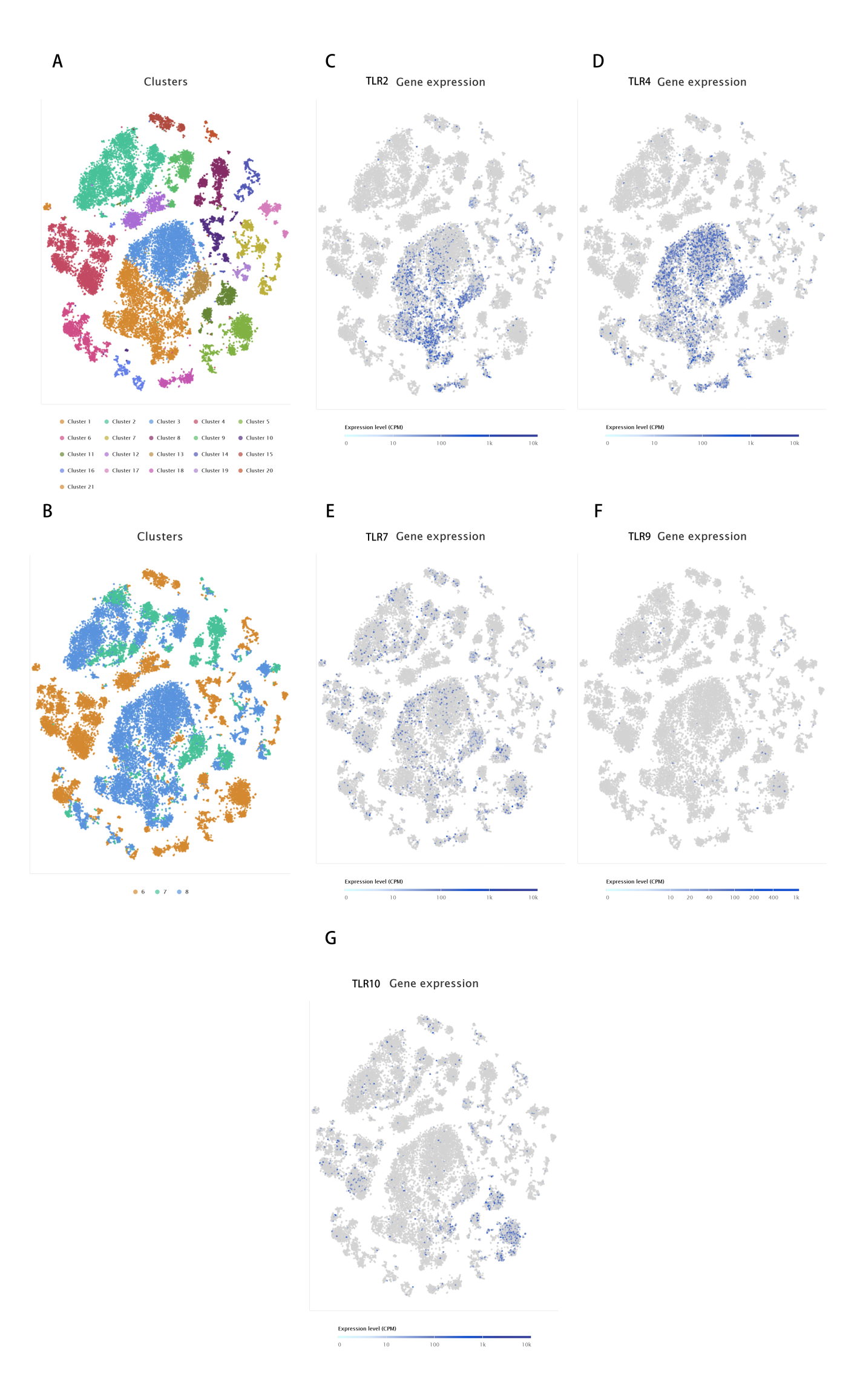


## Figure S11 Single cell sequencing of lung endothelial cells and tumor endothelial cells form NSCLC patients.

## Mixture of cells were divided into 21 clusters.

## Cells from patient 6, 7, and 8 were labeled separately.

## (C-G) Expression of TLR2, TLR4, TLR7, TLR9 and TLR10 at the single cell level.

##
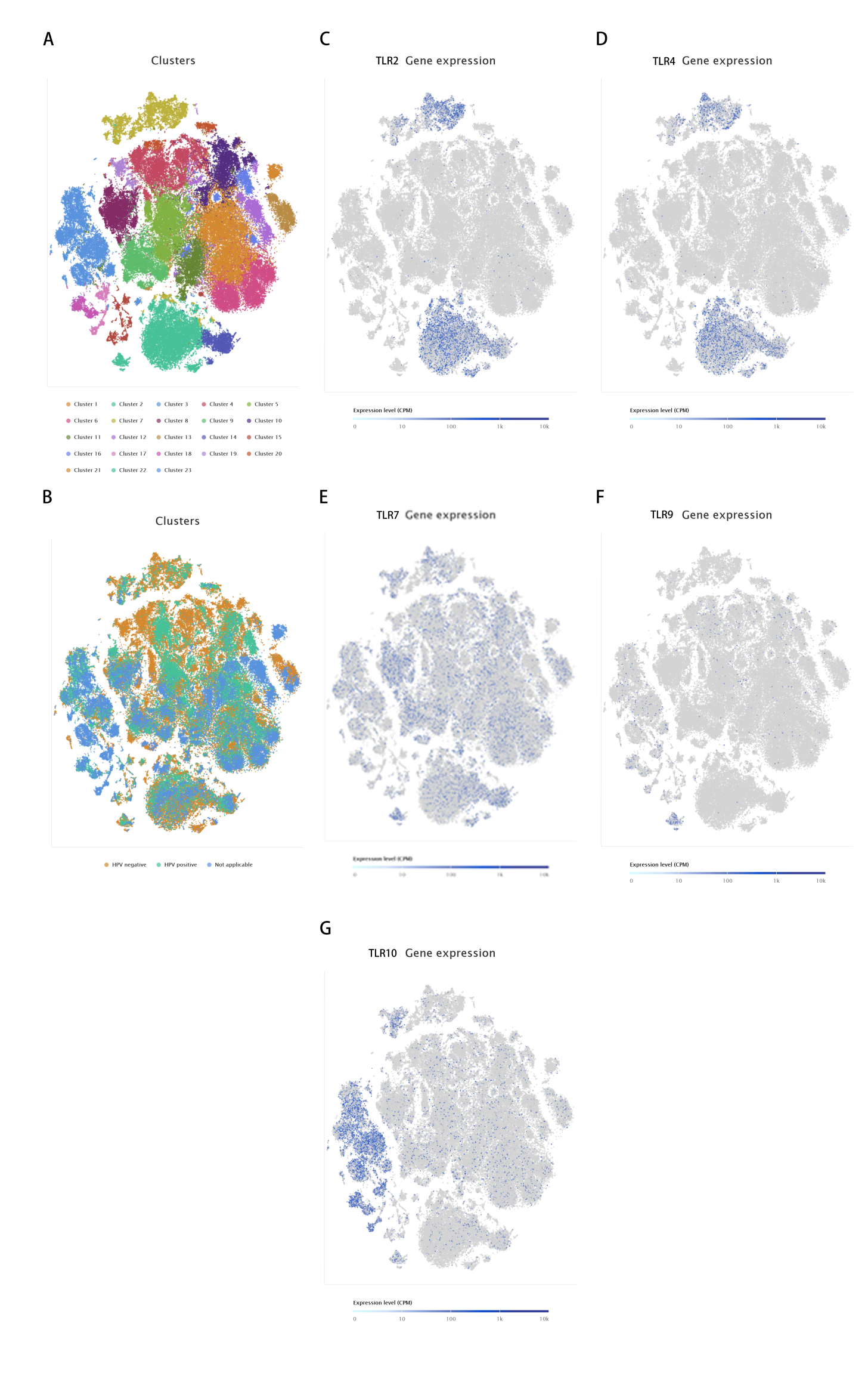


## Figure S12 Single cell sequencing of human head and neck squamous carcinoma tissues and paired normal tissues.

## Mixture of cells were divided into 23 clusters.

## Cells from HPV+ and HPV- HNSCC were labeled separately.

## (C-G) Expression of TLR2, TLR4, TLR7, TLR9 and TLR10 at the single cell level.


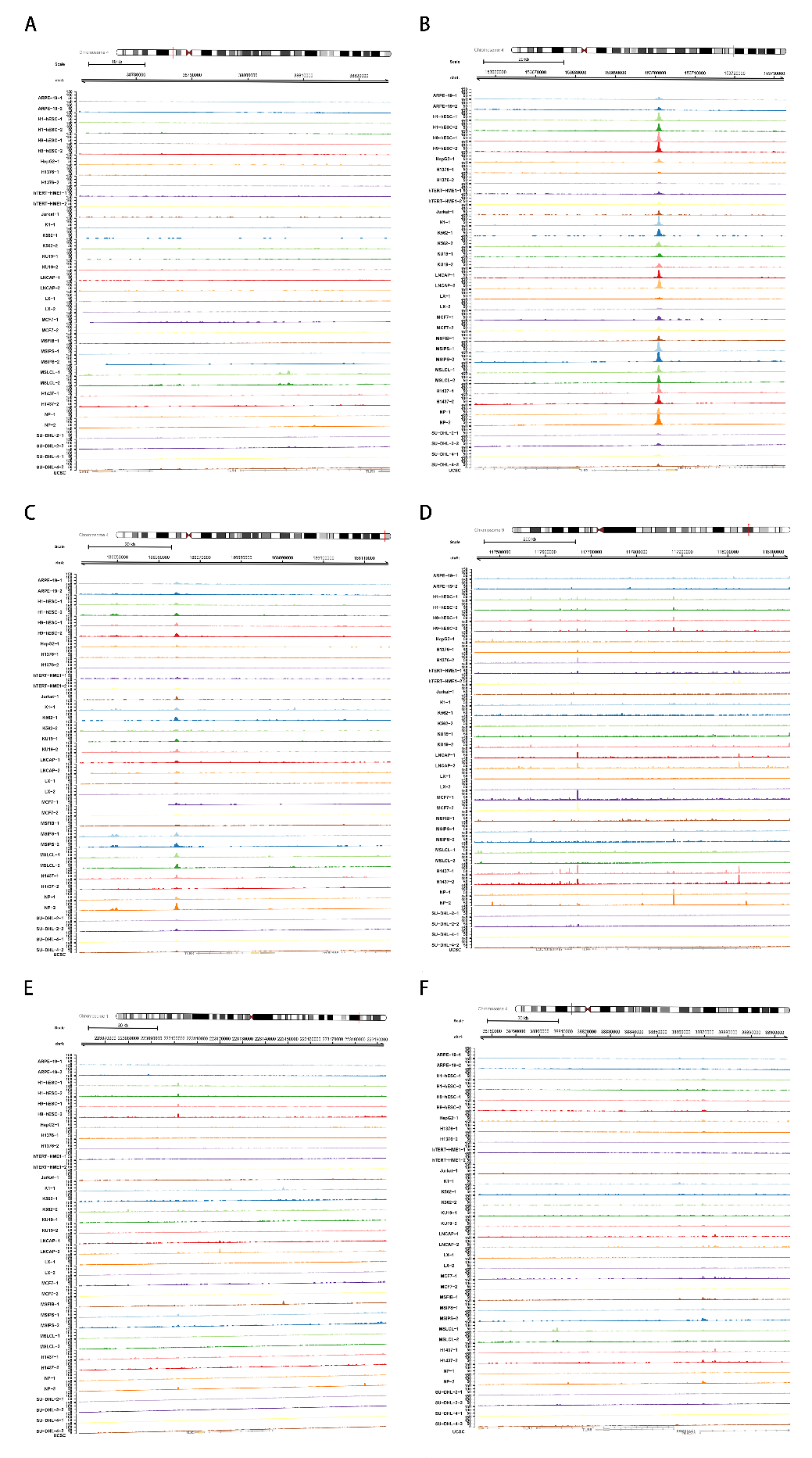


Figure S13 ATAC-Seq peaks in chromatin from TLR1 to TLR6 (A-F) loci in different tumor cell lines.


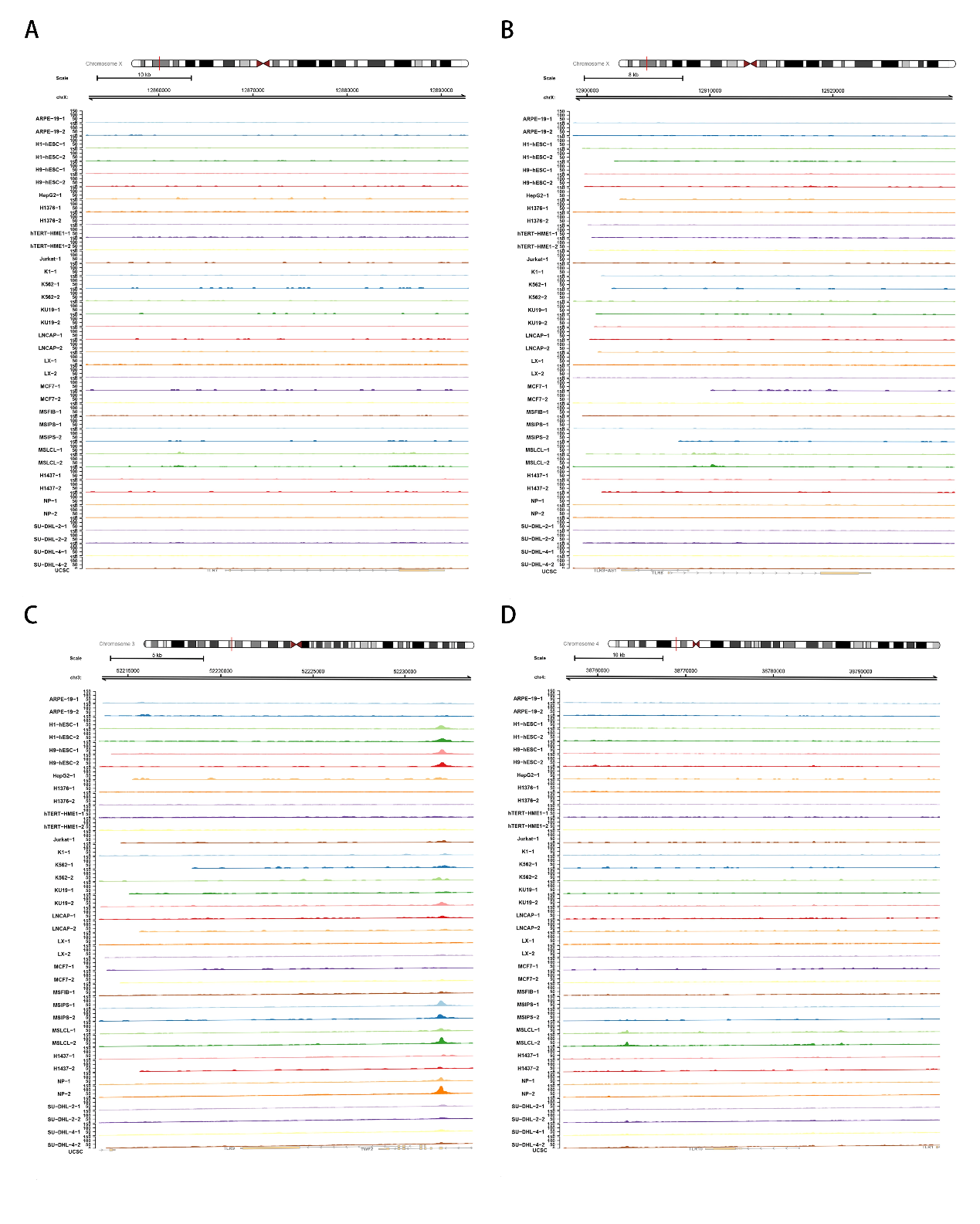


Figure S14 ATAC-Seq peaks in chromatin from TLR7 to TLR10 (A-D) loci in different tumor cell lines.

##
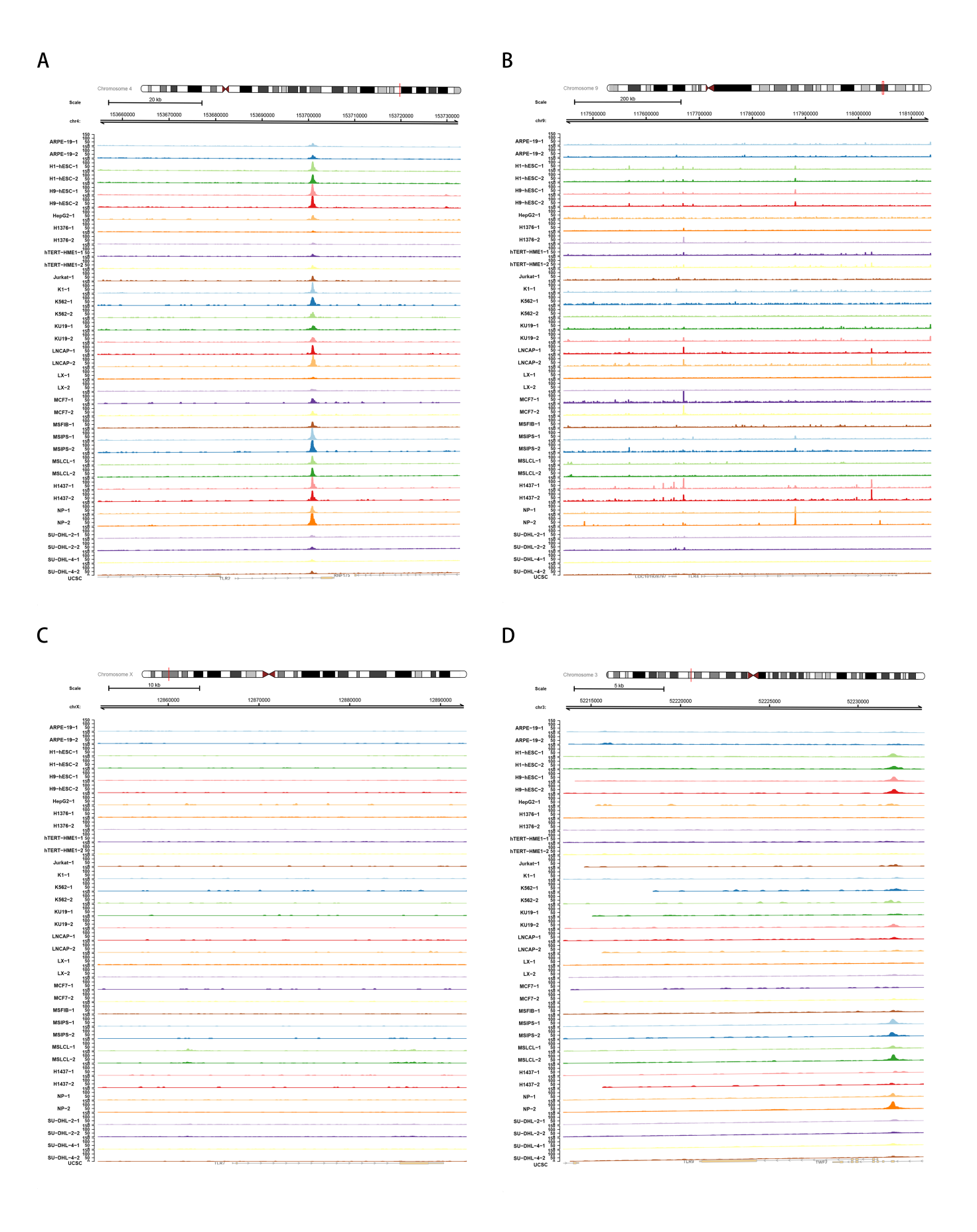


## Figure S15 Chromatin accessibility analysis of TLR family in different neoplasm cell lines. Results of TLR2, TLR4, TLR7 and TLR9 were shown from (A) to (D) respectively.

Table S1 Abbreviation for 33 types of cancer in TCGA database.

| Abbreviation | TCGA Cancer |
| --- | --- |
| ACC | Adrenocortical carcinoma |
| BLCA | Bladder urothelial carcinoma |
| BRCA | Breast invasive carcinoma |
| CESC | Cervical squamous cell carcinoma and endocervical adenocarcinoma |
| CHOL | Cholangiocarcinoma |
| COAD | Colon adenocarcinoma |
| DLBC | Lymphoid neoplasm diffuse large B-cell lymphoma |
| ESCA | Esophageal carcinoma |
| GBM | Glioblastoma multiforme |
| HNSC | Heard and neck squamous cell carcinoma |
| KICH | Kidney chromophobe |
| KIRC | Kidney renal clear cell carcinoma |
| KIRP | Kidney renal papillary cell carcinoma |
| LAML | Acute myeloid leukemia |
| LGG | Brain lower grade glioma |
| LIHC | Liver hepatocellular carcinoma |
| LUAD | Lung adenocarcinoma |
| LUSC | Lung squamous cell carcinoma |
| MESO | Mesothelioma |
| OV | Ovarian serous cystadenocarcinoma |
| PAAD | Pancreatic adenocarcinoma |
| PCPG | Pheochromocytoma and paraganglioma |
| PRAD | Prostate adenocarcinoma |
| READ | Rectum adenocarcinoma |
| SARC | Sarcoma |
| SKCM | Skin cutaneous melanoma |
| STAD | Stomach adenocarcinoma |
| TGCT | Testicular germ cell tumors |
| THCA | Thyroid carcinoma |
| THYM | Thymoma |
| UCEC | Uterine corpus endometrial carcinoma |
| UCS | Uterine carcinosarcoma |
| UVM | Uveal melanoma |

Table S2 Abbreviation for 23 TCGA cancer types in ATAC-Seq research.

| Abbreviation | TCGA Cancer in ATAC-Seq research |
| --- | --- |
| ACC | Adrenocortical carcinoma |
| BLCA | Bladder urothelial carcinoma |
| BRCA | Breast invasive carcinoma |
| CESC | Cervical squamous cell carcinoma and endocervical adenocarcinoma |
| CHOL | Cholangiocarcinoma |
| COAD | Colon adenocarcinoma |
| ESCA | Esophageal carcinoma |
| GBM | Glioblastoma multiforme |
| HNSC | Heard and neck squamous cell carcinoma |
| KIRC | Kidney renal clear cell carcinoma |
| KIRP | Kidney renal papillary cell carcinoma |
| LGG | Brain lower grade glioma |
| LIHC | Liver hepatocellular carcinoma |
| LUAD | Lung adenocarcinoma |
| LUSC | Lung squamous cell carcinoma |
| MESO | Mesothelioma |
| PCPG | Pheochromocytoma and paraganglioma |
| PRAD | Prostate adenocarcinoma |
| SKCM | Skin cutaneous melanoma |
| STAD | Stomach adenocarcinoma |
| TGCT | Testicular germ cell tumors |
| THCA | Thyroid carcinoma |
| UCEC | Uterine corpus endometrial carcinoma |
